# Long-distance transport of sucrose in source leaves promotes sink root growth by the EIN3-SUC2 module

**DOI:** 10.1371/journal.pgen.1010424

**Published:** 2022-09-21

**Authors:** Chen Tong, Cong Li, Xiao-Ying Cao, Xu-Dong Sun, Qin-Xin Bao, Xin-Rong Mu, Chang-Yue Liu, Gary J. Loake, Hu-hui Chen, Lai-Sheng Meng

**Affiliations:** 1 School of Life Science, Jiangsu Normal University, Xuzhou, Jiangsu, People’s Republic of China; 2 Public Technical Service Center, Kunming Institute of Zoology, Chinese Academy of Sciences, Kunming, Yunnan, People’s Republic of China; 3 Kunming Institute of Botany, Chinese Academy of Sciences, Kunming, Yunnan, People’s Republic of China; 4 Jiangsu Normal University - Edinburgh University, Centre for Transformative Biotechnology of Medicinal and Food Plants, Jiangsu Normal University, Xuzhou, People’s Republic of China, China; 5 Institute of Molecular Plant Sciences, School of Biological Sciences, Edinburgh University, King’s Buildings, Edinburgh, United Kingdom; 6 School of Life Science, Nanjing Agricultural University, Nanjing, Jiangsu, People’s Republic of China; Wake Forest University, UNITED STATES

## Abstract

In most plants, sucrose, a major storage sugar, is transported into sink organs to support their growth. This key physiological process is dependent on the function of sucrose transporters. Sucrose export from source tissues is predominantly controlled through the activity of SUCROSE TRANSPORTER 2 (SUC2), required for the loading of sucrose into the phloem of *Arabidopsis* plants. However, how SUC2 activity is controlled to support root growth remains unclear. Glucose is perceived via the function of HEXOKINASE 1 (HXK1), the only known nuclear glucose sensor. HXK1 negatively regulates the stability of ETHYLENE-INSENSITIVE3 (EIN3), a key ethylene/glucose interaction component. Here we show that *HXK1* functions upstream of *EIN3* in the regulation of root sink growth mediated by glucose signaling. Furthermore, the transcription factor EIN3 directly inhibits SUC2 activity by binding to the *SUC2* promoter, regulating glucose signaling linked to root sink growth. We demonstrate that these molecular components form a HXK1-EIN3-SUC2 module integral to the control of root sink growth. Also, we demonstrate that with increasing age, the HXK1-EIN3-SUC2 module promotes sucrose phloem loading in source tissues thereby elevating sucrose levels in sink roots. As a result, glucose signaling mediated-sink root growth is facilitated. Our findings thus establish a direct molecular link between the HXK1-EIN3-SUC2 module, the source-to sink transport of sucrose and root growth.

## Author summary

In *Arabidopsis* and most crops, sucrose transporters are positioned in the vascular bundles of leaf blades where they have crucial roles in balancing source and sink activities. However, little molecular detail is currently available regarding the modulation of sucrose transporter activity. Here, we demonstrate that the transcriptional regulator, *EIN3*, functions downstream of HEXOKINASE 1 (HXK1), which acts upstream of *SUC2* in the regulation of root sink growth mediated by glucose signaling. Further, EIN3 directly represses SUC2 function by negatively regulating *SUC2* transcription. We further demonstrate that these components form the HXK1-EIN3-SUC2 module to facilitate sucrose phloem loading in source tissues thereby elevating sucrose content in sink roots.

## Introduction

Sugars produced from photosynthesis affect plant growth and development throughout the life cycle, from germination to senescence. Sucrose is a major photosynthetic product and the predominant transported sugar [1]. The loading of sucrose into the phloem is essential for *Arabidopsis* growth, development and reproduction. The sucrose transporter SUC2 in *Arabidopsis* is a phloem-specific SUC (SUCROSE TRANSPORTER), which is specifically expressed in companion cells [2]. SUC2 has a central role in the loading of sucrose into the phloem and is essential for high-performance sucrose transport from source to sink organs in *Arabidopsis* [2–4]. The *suc2* mutant exhibits stunted growth, delayed development and sterility. Further, these mutant plants accumulate massive amounts of starch and sucrose in the leaf blades and transport of sugar to sink organs, including roots, the shoot apex and inflorescences is blocked [3]. Therefore, SUC2, is a key sucrose transporter that mediates sucrose phloem transport and its loss-of-function leads to seriously delayed growth and thereby accumulated sucrose in source tissues. A previous report has shown that growth of sink roots may be promoted by exogenously provided glucose acting through either signaling or metabolism [5]. However, whether and how plant root growth is mediated by the loading of sucrose into the phloem is largely unknown.

In plants, sucrose cannot be directly utilized, rather it is irreversibly hydrolyzed by invertase to the hexoses, glucose and fructose [3]. Glucose is perceived and transduced through two principal mechanisms: indirect sensing via a variety of energy and metabolite sensors including [SNF1-RELATED PROTEIN KINASE1 (SnRK1) and target of rapamycin (TOR)] or direct sensing through glucose sensors [6]. Glucose is indirectly sensed by the cellular energy sensor, SnRK1 (SNF1-RELATED KINASE1), which in turn directly interacts with and subsequently phosphorylates and promotes the degradation of a key ethylene signaling component, ETHYLENE-INSENSITIVE3 (EIN3), a plant-specific transcription factor [6]. On the other hand, the direct glucose sensor, Hexokinase1 (HXK1), has both signaling and enzymatic functions [7–8] and is the only known nuclear glucose sensor [7]. Glucose indirectly and negatively controls the stability of the EIN3 transcriptional regulator via HXK1, whereas the mechanistic link between glucose sensing and EIN3 degradation has not to date been demonstrated [9]. EIN3 stability rapidly increases with ethylene treatment, while in the absence of ethylene signaling, EIN3 is targeted via the SCF^EBF1/EBF2^ and subsequently degraded via the 26S proteasome [10–11]. Through binding to specific promoter element termed an EIN3 binding site (EBS), EIN3 modulates the expression of numerous downstream target genes.

In this study, we show that glucose decreases the stability of EIN3 by the activity of a glucose sensor HEXOKINASE 1, which in turn elevates SUC2 function within source tissues, enhancing the loading of sucrose into the phloem in these tissues and thereby promoting the growth of sink organs, such as roots.

## Results

### *EIN3* and *EIL1*, acting downstream of *HXK1*, negatively regulate glucose signaling mediated-root growth

**Fig 1:**
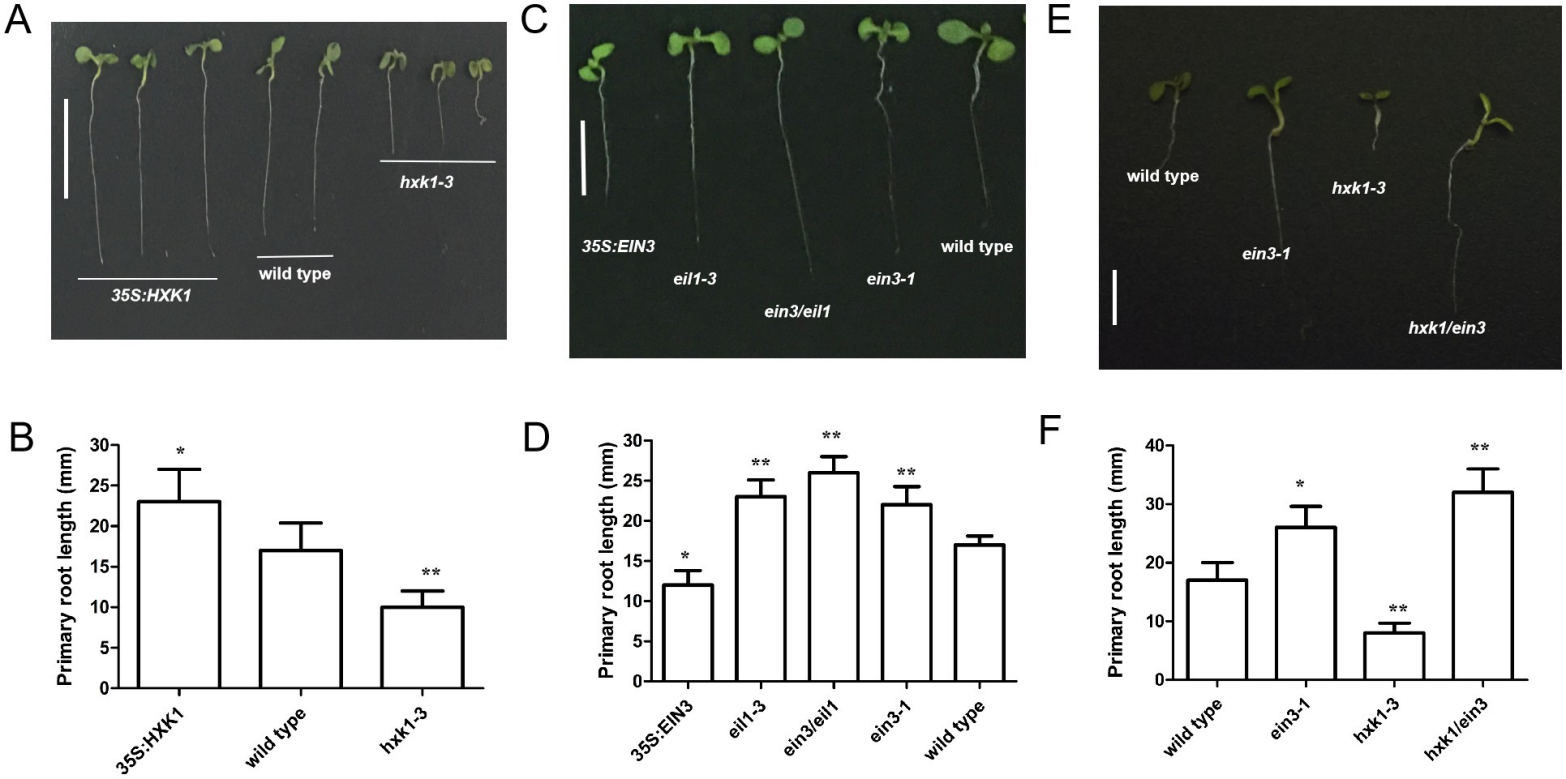
*HXK1* is upstream of *EIN3/EIL1* to positively regulate primary root growth. **A**, The image illustrating 5-day-old *Arabidopsis 35S*:*HXK1*, wild-type and *hxk1-3* seedlings grown on MS medium with 1% glucose. Bar = 1.0 cm. **B**, Bar graph illustrating the differences in primary root length in (A). **C**, The image illustrating 5-day-old *35S*:*EIN3*, *eil1-3*, *ein3/eil1*, *ein3-1* and wild-type seedlings grown on MS medium with 1% glucose. Bar = 1.0 cm. **D**, Bar graph illustrating the differences in primary root length in (C). **E**, The image illustrating 5-day-old *hxk1-3*, *ein3-1* and *hxk1/ein3* seedlings grown on MS medium with 1% glucose. Bar = 1.0 cm. **F**, Bar graph illustrating the differences in primary root length in (E). Error bars represent SD (n = 12). Student’s *t* test (**P < 0.01; *P < 0.05).

Previous data has shown that root growth may be promoted through glucose [5]. Glucose is directly perceived by the function of HXK1, the only known nuclear glucose sensor. Therefore, we explored if HXK1 was involved in the regulation of root growth. Whereas the *HXK1/GIN2* loss-of-function mutant, *gin2-1*, is in the L*er* background, another loss-of-function mutant, *hxk1-3*, is in the Col-0 background. When *Arabidopsis* seedlings were grown on solid MS medium with 1% glucose, both the *hxk1-3* and *gin2-1* mutants exhibited a shorter primary root length, compared to wild-type seedlings (Fig 1A and 1B and S1 Fig). By contrast, corresponding seedlings expressing a cauliflower mosaic virus *35S (35S)*:*HXK1* transgene showed elongated primary root length compared to wild-type (Fig 1A and 1B). These findings indicate that *HXK1* promotes root growth.

Glucose indirectly and negatively controls the stability of the EIN3 transcriptional regulator through HXK1 function [7]. Therefore, we determined if EIN3/EIL1 is involved in the regulation of root growth. We employed *Arabidopsis* plants expressing a cauliflower mosaic virus *35S* (*35S*):*EIN3* transgene (S2A Fig) and loss-of-function mutations in either *EIN3* or the most closely related *EIN3* paralog, *EIN3-like 1* (*EIL1*) [12], to investigate this posit. When *35S*:*EIN3*, *eil1-3*, *ein3/eil1*, *ein3-1* and wild-type seedlings were grown on solid MS medium without sugars for 5 days, their primary root length was not significantly different (S3 Fig). However, when these seedlings were grown on solid MS medium with 1% glucose for 5 days, the *35S*:*EIN3* line showed a shortened primary root length, compared to wild-type (Fig 1C and 1D and S4B and S4D Fig). In contrast, *eil1-3*, *ein3/eil1* and *ein3-1* seedlings showed elongated roots relative to wild-type (Fig 1C and 1D and S4A and S4C Fig). These findings indicate that *EIN3* inhibits root growth.

To determine whether *EIN3* acts downstream of *HXK1* in the regulation of root growth, we crossed *hxk1-3* with *ein3-1* plants. The *ein3-1* line generated elongated primary roots, compared with wild-type plants (Fig 1E and 1F). Further, *hxk1/ein3* mutant plants also generated elongated primary roots, similar to that of the *ein3-1* mutant (Fig 1E and 1F). These findings indicate that *EIN3* acts downstream of *HXK1* in the regulation of root growth.

**Fig 2:**
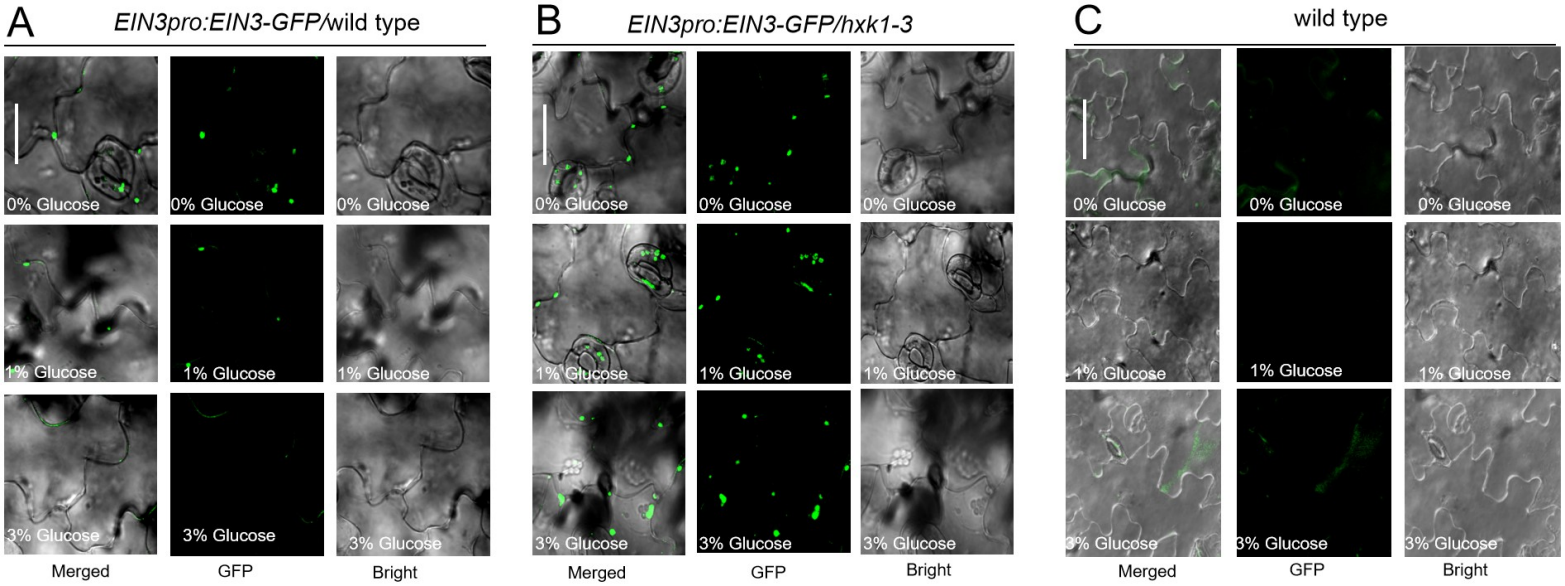
Transiently applied glucose suppresses EIN3, dependent on HXK1 function. **A, B** and **C**, Images illustrating the nuclear localization of EIN3pro:EIN3-GFP in leaf blades of 5-day-old *EIN3pro*:*EIN3-GFP* in wild-type (A) and *hxk1-3* (B) background. Wild type was used as negative control (C). These seedlings were grown on MS medium with 1%, 3% glucose or without for 5 days. Bar = 20 μm. Three biological replicates were analyzed for each pair and one representative result was presented.

To investigate the link between glucose signaling and EIN3 dependent signaling, we generated a *EIN3pro*:*EIN3-GFP/hxk1-3* line. A decrease of *EIN3pro*:*EIN3-GFP* signaling in wild-type, but not in the *hxk1-3* line was apparent post glucose application (Fig 2). Thus, increased GFP fluorescence was generated in the *hxk1-3* relative to that than in the wild-type background. These findings indicate that glucose decreases EIN3 stability via the glucose signaling sensor, HXK1.

Taken together, our results show that HXK1 positively regulates and EIN3 negatively regulate primary root growth. Further, *EIN3* act downstream of *HXK1* in the regulation of glucose signaling that mediates root growth.

### EIN3 negatively regulates glucose signaling mediated-expression of *SUC2*

Chromatin immunoprecipitation sequence (ChIP-Seq) analysis of EIN3 [13] indicates that EIN3 may directly regulate expression of the *Sucrose Transporter 4* (*SUC4*) [14]. Further, SUC2, but not SUC4, has been proposed to function as a major transporter of sucrose loading into the phloem in *Arabidopsis* [1]. Therefore, we determined if *SUC2* is a downstream target gene of EIN3.

**Fig 3:**
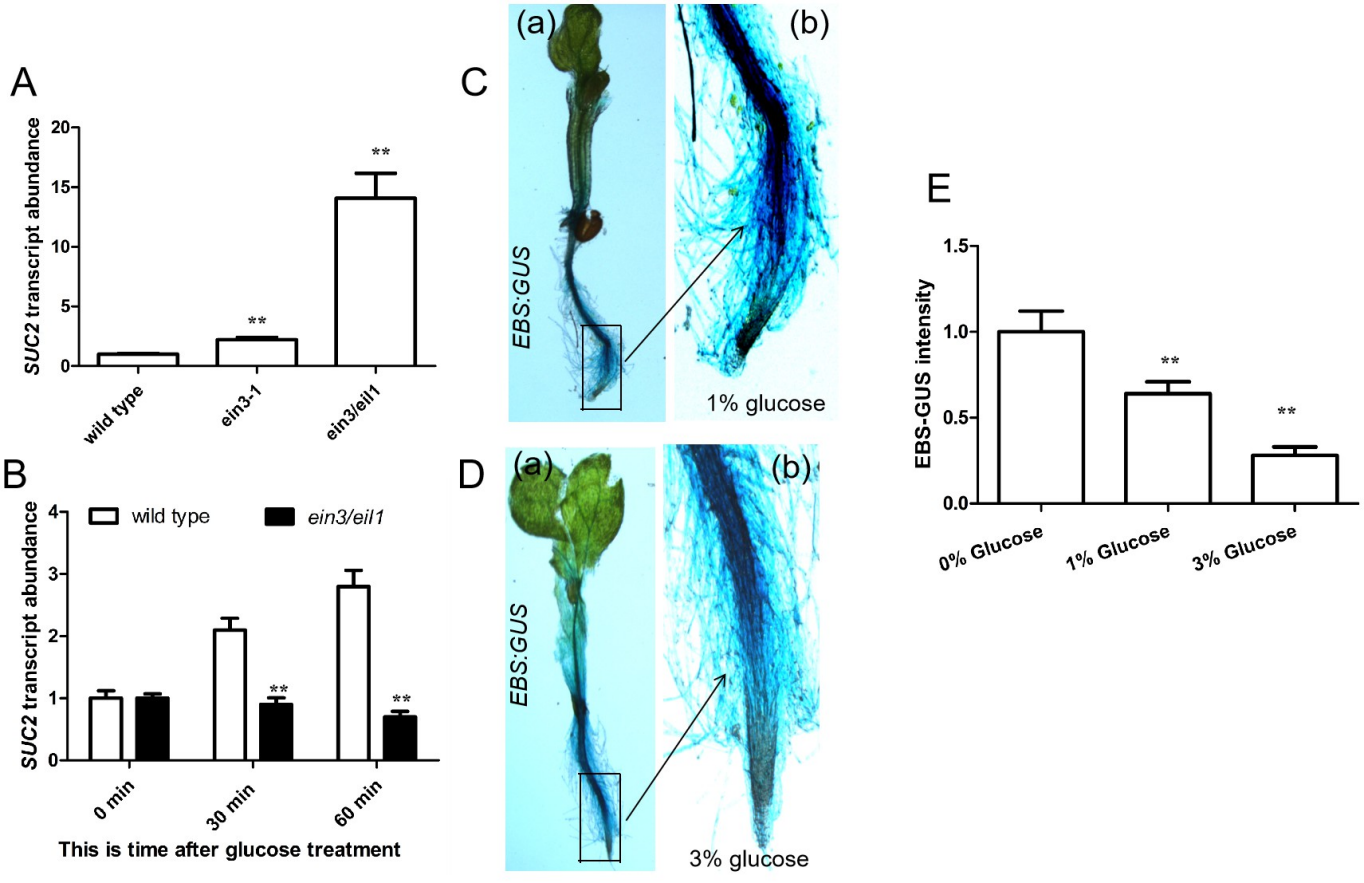
EIN3 negatively regulates *SUC2* expression. **A**, Bar graph showing the differential expression of *SUC2* among 5-day-old wild-type, *ein3-1* and *ein3/eil1* seedlings grown on MS medium with 1% glucose. Quantification of the expression of the *SUC2* gene in wild-type seedlings was set as 1.0. **B**, Bar graph showing the differences in the expression level of *SUC2* between wild-type and *ein3/eil1* seedlings with different time treatment. Wild-type seeds were sowed and grown in soil for 10 days. And then these seedlings were gathered and treated with 2% glucose for 30 min and 60 min. Total RNA was extracted from these treated seedlings and qPCR was performed. Quantification of 10-day-old wild-type seedlings without treatment (control) is set as 1.0 in B. **C** and **D**, Images showing seedlings of *EBS* (*EIN3 binding site*)*-GUS* were grown on solid MS medium with 1% glucose (C) or 3% glucose (D) for 4 days in light, and then *GUS* staining was performed. (b) is amplified from the rectangle indicated in A/B (a). **E**, Bar graph showing intensity of *EBS-GUS* (C and D) quantified using Adobe Photoshop CS (Adobe Systems Inc., San Jose, CA) software, as described by Meng and Yao (2015). Quantification of 0% glucose is set as 1.0. Quantifications were normalized to *UBQ5* expression in A and B. Error bars represent SD (n = 3). Student’s *t* test (**P < 0.01).

We first performed quantitative (q)PCR experiments to monitor the transcript abundance of *SUC2* in wild-type, *ein3-1* and *ein3/eil1* seedlings. When seedlings were grown on MS medium with 1% glucose for 5 days, the expression of *SUC2* was significantly elevated in *ein3-1* and *ein3/eil1* plants relative to wild-type (Fig 3A). These qPCR results are consistent with previously reported data [15]. These findings indicate that EIN3 might repress *SUC2* expression. Further, we monitored *SUC2* expression over time in response to exogenously supplied glucose. The expression of *SUC2* in wild-type, but not *ein3/eil1* seedlings, was increased post glucose application (Fig 3B), indicating that glucose signaling promoted *SUC2* expression is partially dependent on EIN3 and EIL1.

To monitor EIN3-dependent transcriptional activity during *Arabidopsis* root growth, we employed a transgenic line containing the *b-glucoronidase* (*GUS* reporter gene) driven by five tandem repeats of the EBS driven by a minimal *35S* promoter (*5 × EIN3 (EIN3 binding site)-GUS*) [16]. GUS staining intensity in roots decreased with increasing exogenous glucose (Fig 3C–3E), suggesting that glucose decreases the transcriptional activity of EIN3.

Together, these findings suggest that with increasing age, glucose accumulation decreases the transcriptional activity of EIN3, which may in turn elevate glucose signaling mediated-expression of *SUC2*.

### EIN3 directly binds to the promoter of *SUC2* to repress *SUC2* expression

**Fig 4:**
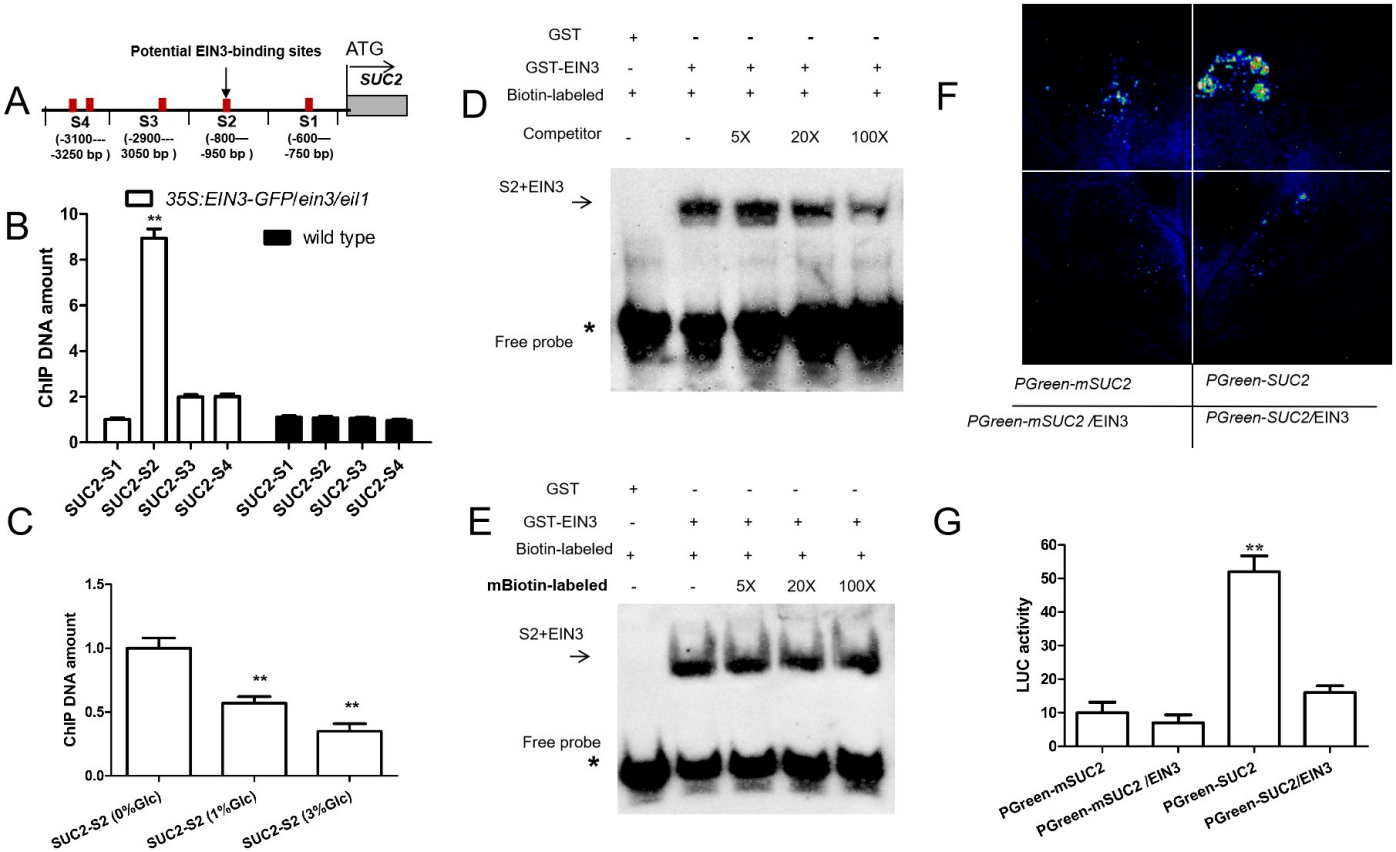
EIN3 directly interacts with promoter of *SUC2* to suppress its expression. **A**, Schematic of the promoter loci of *SUC2* and their amplicons for ChIP analysis. **B**, Bar graph illustrating chromatin immunoprecipitation (ChIP) analysis. Enrichment of particular chromatin regions of *SUC2* with anti-GFP antibody in *35S*:*EIN3-GFP/ein3/eil1* and wild-type seedlings grown in soil for 25 days, as detected by real-time PCR analysis. Quantifications were normalized to the expression of *UBQ5*. **C**, Bar graph illustrating ChIP-PCR in glucose treatment. Ten-day-old *35S*:*EIN3-GFP/ein3/eil1* seedlings grown in soil. And then these wild-type seedlings were treated by using 0%, 1% and 3% glucose for 30 mins. The ChIP DNA amount at 0% glucose for 30 mins is set as 1.0. Quantification was normalized to the expression of *UBQ5*. **D**, Image illustrating unlabeled promoter of *SUC2* were used as competitors to determine the specificity of DNA-binding activity for EIN3. **E**. Image illustrating a mutant version of the promoter of *SUC2* was labeled with biotin and used for EMSA with EIN3 polypeptides. Free probe and EIN3 probe complexes are indicated by an asterisk and arrows, respectively in D and E. **F**, Image illustrating luminescence units is represented by LUC activity. **G**, Bar graph illustrating transient expression of the 35S:EIN3 effector construct with the *SUC2pro-LUC* reporter construct in *N*. *benthamiana* leaves. Note *PGreen-mSUC2* indicates the conserved sites (TTCAAA) of the S2 region in *SUC2* promoter were mutated. The activity of relative LUC represents arbitrary luminescence units, that is, expressing *SUC2-LUC* is ~10, other expressions was quantified by using Adobe Photoshop CS (AdobeSystems) software, as described previously by [28]. Error bars represent SD (n = 3). Student’s *t* test (**P < 0.01).

Investigation of the DNA sequence comprising the promoter of *SUC2* revealed a few putative EIN3 binding sites (EBSs) [16] (Fig 4A). To explore if EIN3 can interact with any of these EBSs, we employed a transgenic *Arabidopsis* line possessing a *35S*:*EIN3-GFP* transgene in an *ein3/eil1* genetic background (*35S*:*EIN3-GFP/*wild-type crossed with *ein3/eil1* and subsequently *35S*:*EIN3-GFP/ein3/eil1* seedlings were identified by qPCR). We subsequently utilized chromatin immunoprecipitation (ChIP) analysis to determine whether the transcription factor EIN3 directly binds to the EBSs in the promoter sequence of *SUC2*. Indeed, in the absence of glucose, EIN3 directly bound to one *SUC2* promoter sequence (S2), but not to three other *SUC2* promoter sequences (S1, S3 and S4 Figs) (Fig 4B). Further, glucose treatment decreased the amount of amplified S2 sequence of the *SUC2* promotor after an anti-GFP ChIP (Fig 4C), which may result from glucose decreasing EIN3 transcriptional activity (Fig 3C–3E).

To confirm EIN3 could directly bind to the S2 EBS within the promoter of *SUC2 in vitro*, electrophoretic mobility shift assay (EMSA) experiments were performed. Indeed, EIN3 bound exclusively to the labeled S2 EBS element *in vitro* (Fig 4D). Excess unlabeled competitor DNA abolished this binding ability, implying EIN3 binding specificity (Fig 4D). Moreover, EIN3 binding to the labeled S2 EBS element *in vitro* was not abolished by the mS2 (mutant S2) DNA sequence *in vitro* (Fig 4E). Further, transient expression data in *N*. *benthamiana* showed that co-expressions of *EIN3* decreased the expression of the reporter gene *PGreenSUC2pro*:*Lucifertase (LUC)* (Fig 4F and 4G).

Taken together, these data indicate that EIN3 directly binds to the *SUC2* promoter to suppress *SUC2* expression.

### *SUC2* acts downstream of *EIN3* to positively regulate root growth

Since EIN3 negatively regulates root growth (Fig 1) and EIN3 directly inhibits the expression of *SUC2* (Figs 3 and 4), we therefore determined if SUC2 positively regulates root growth.

**Fig 5:**
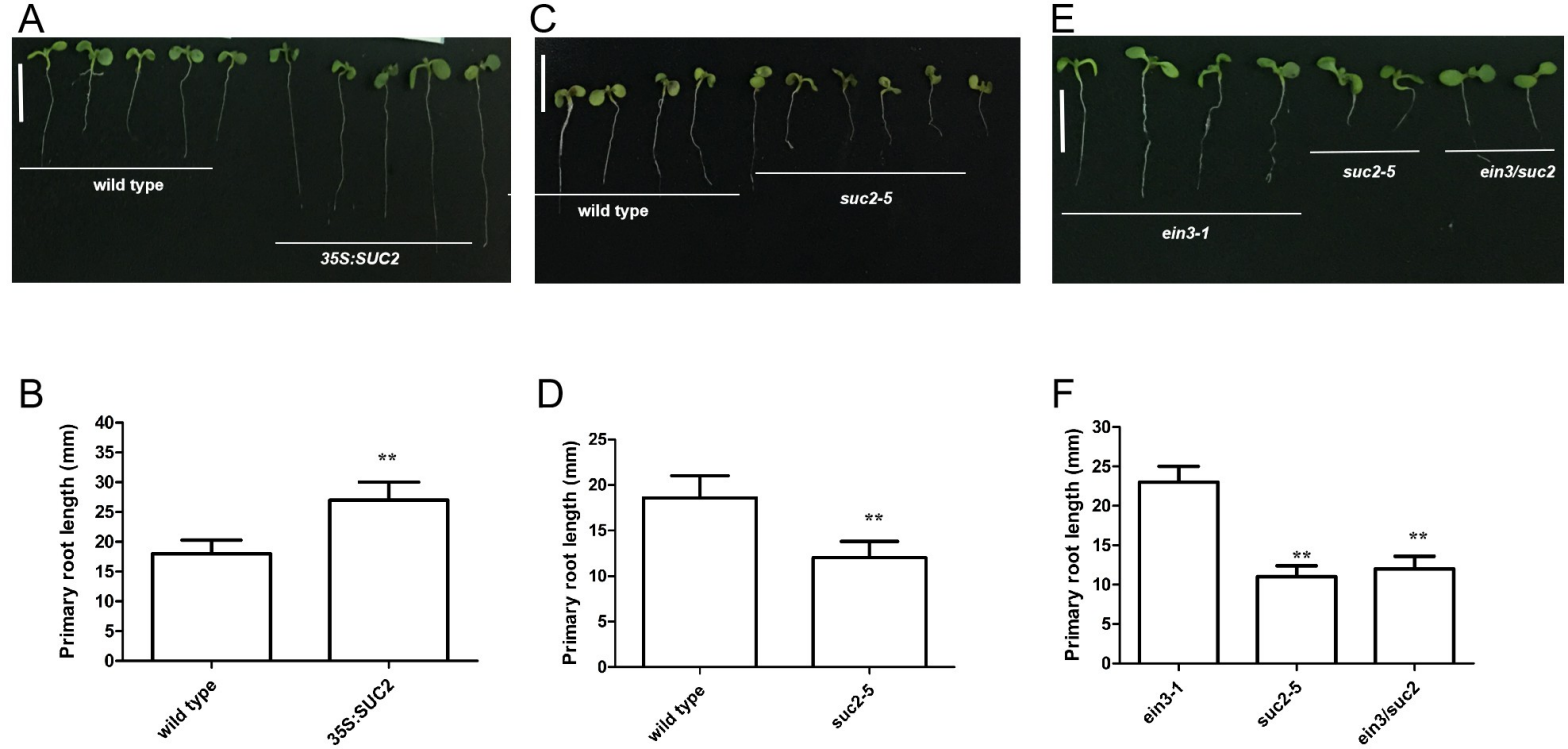
*SUC2* is downstream of *EIN3* to positively regulate primary root growth. **A**, Image illustrating the 5-day-old *Arabidopsis* wild-type and *35S*:*SUC2* seedlings grown on MS medium with 1% glucose. Bar = 1.0 cm. **B**, Bar graph illustrating the differences in primary root length in (A). **C**, Image illustrating the 5-day-old wild-type and *suc2-5* seedlings grown on MS medium with 1% glucose. Bar = 1.0 cm. **D**, Bar graph illustrating the differences in primary root length in (C). **E**, Image illustrating the 5-day-old *ein3-1*, *suc2-5* and *ein3/suc2* seedlings grown on MS medium with 1% glucose. Bar = 1.0 cm. **F**, Bar graph illustrating the differences in primary root length in (E). Error bars represent SD (n = 14). Student’s *t* test (**P < 0.01).

In short days, *Arabidopsis* plants expressing a cauliflower mosaic virus *35S* (*35S*):*SUC2* transgene (S2B Fig) exhibited elongated primary root length, compared to wild-type seedlings (Fig 5A and 5B and S5A and S5C Fig). By contrast, *SUC2* loss-of-function homozygous *suc2-5* (*suc2-5* is a null allele) mutant seedlings exhibited severely retarded growth and development [17]. We observed that seedlings of *suc2-5* showed a shortened primary root length, compared to wild-type (Fig 5C and 5D and S5B and S5D Fig). These findings indicate that *SUC2* promotes root growth.

To determine whether *EIN3* genetically acts upstream of *SUC2* in the regulation of root growth, we crossed *suc2-5* with *ein3-1* seedlings. The *ein3-1* line exhibited elongated primary roots, whereas *suc2-5* seedlings produced a shortened primary root, compared with wild-type (Fig 5E and 5F). Further, *ein3/suc2* mutant seedlings generated a shortened primary root, which was similar to that of the *suc2-5* mutant (Fig 5E and 5F).

Together, these findings indicate that *SUC2* genetically acts downstream of *EIN3* to positively regulate root growth.

### Sucrose phloem loading and accumulation in source and sink organs are regulated by an EIN3-SUC2 module

In *Arabidopsis*, *SUC2* encodes a proton/sucrose symporter localized throughout collection and transport phloem within the vasculature and is thus essential for effective transport of sucrose from source to sink organs [4]. Therefore, we determined if the EIN3-SUC2 module mediates the loading of sucrose into the phloem.

**Fig 6:**
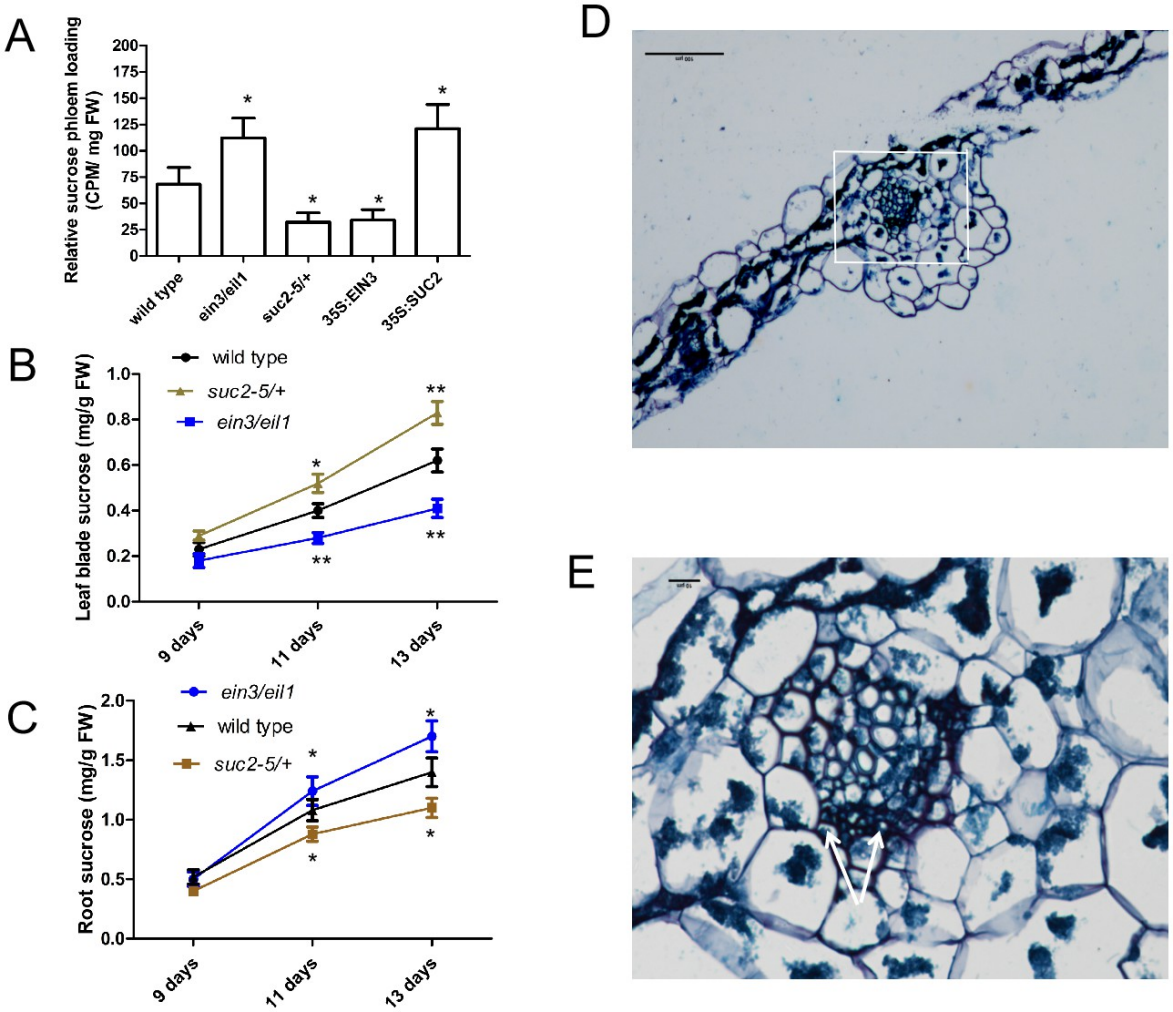
Sucrose phloem loading of mature leaves or shoot apex and sucrose levels from a few lines. **A**, Phloem loading of sucrose in wild-type, *ein3/eil1*, *suc2-5/+*, *35S*:*EIN3* and *35S*:*SUC2* vegetative leaves. These seedlings were grown in soil for 15 days under short days and normal light conditions, and vegetative leaves were immersed in the sucrose phloem loading buffer with [^14^ C] sucrose and incubated for 3 hours. The amount of [^14^C] sucrose phloem loading was assayed via a scintillation counter. FW, Fresh weight. **B**, Levels of vegetative leaf sucrose (source tissue) in 9-, 11- and 13-day-old *ein3/eil1*, *suc2-5/+* and wild-type plants grown in soil at exposure to light for 4 hours. Sucrose per gram fresh weight. **C**, Levels of root sucrose (sink tissue) in 9-, 11- and 13-day-old *ein3/eil1*, *suc2-5/+* and wild-type plants grown in soil at exposure to light for 8 hours. Sucrose per gram fresh weight. Error bars represent SD (n = 3). Student’s *t* test (**P < 0.01; *P < 0.05). **D and E**, The expression of vegetative leaves in *5×EBS* (*EIN3 binding site*)-*GUS* seedlings. Parafin section of leaf blades. Arrows indicate sieve tubes and companion cells in phloems in D. Bars = 100 μm in D and E. E is amplified in the pane of D.

A [^14^C] sucrose phloem loading assay showed that the rate of sucrose phloem loading in wild-type, *ein3/eil1*, *suc2-5/+*, *35S*:*EIN3* and *35S*:*SUC2* seedlings was significantly changed (Fig 6A) (for a detailed procedure of measuring sucrose phloem loading rate, see “Materials and Methods”). Thus, the sucrose phloem loading rate was significantly elevated in *ein3/eil1* and *35S*:*SUC2* seedlings, whereas it was decreased in *suc2-5/+* and *35S*:*EIN3* plants (Fig 6A). Since glucose raises SUC2 activity by the EIN3-SUC2 module to promote root growth and glucose/ethylene has antagonistic functions in the regulation of plant growth [9], we determined if ethylene inhibits SUC2 activity. Indeed, the exogenously supplied ethylene precursor 1-aminocyclopropane-1-carboxylic acid (ACC) decreased SUC2 activity (S6 Fig).

To further elucidate EIN3 mediated sucrose phloem loading, we employed a transgenic line containing the *GUS* reporter gene downstream of five tandem repeats of the EBS (*5×EBS*:*GUS*), which has been utilized to check EIN3 transcriptional activity [16]. We observed that EIN3 transcriptional activity was present in the veins of mature leaves of the *5×EBS*:*GUS* line and was also observed in sieve tubes and companion cells in the phloem (Fig 6D and 6E).

To confirm sucrose phloem loading was linked to the EIN3-SUC2 module, we assayed the sucrose level of vegetative leaves (source organs) and roots (sink organs) in 9, 11 and 13 day-old wild-type, *suc2-5/+* and *ein3/eil1* seedlings following exposure to light for 4 and 8 hours, respectively. In all seedlings, sucrose accumulation in both source and sink organs increased with increasing age (Fig 6B and 6C), suggesting total sucrose levels increase with tissue age under these conditions. Further, following exposure to light for 4 hours, sucrose accumulation over time was lower in the *ein3/eil1* vegetative leaves and was higher in the *suc2-5/+* line relative to wild-type (Fig 6B), because the phloem loading rate of sucrose in vegetative leaves over time was higher in the *ein3/eil1* line and lower in *suc2-5/+* plants relative to in wild-type (Fig 6A). As a result, following exposure to light for 8 hours, sucrose accumulation over time was higher in *ein3/eil1* roots and was lower in the *suc2-5/+* line relative to wild-type (Fig 6C), which lead to elongated roots in *ein3/eil1* seedlings and shortened roots in *suc2-5/+* mutant seedlings (Fig 5).

Together, these findings indicate that the EIN3-SUC2 module regulates sucrose accumulation in both vegetative leaves (source organs) and roots (sink organs) by mediating the loading of sucrose into the phloem. As a result, sink growth in roots is promoted by sucrose transport.

## Discussion

Plants acquire their carbon from atmospheric carbon dioxide. During daytime plant sugar phosphates are largely generated via photosynthetic assimilation and are further metabolized to support plant growth and development [1, 2, 3]. Excess photoassimilates are stored mostly in a form of starch in *Arabidopsis* and at relatively low levels of sucrose, both starch and sucrose are in part metabolized via their turnover (synthesis and breakdown) during the day and are stored at night [1, 2, 3]. Under advantageous environmental conditions, enhanced photosynthesis, will in turn enhance sucrose phloem loading to maintain the balance between source and sink [1]. In whole-plant carbon partitioning, it is noteworthy that sucrose phloem loading in source leaf blades is a step.

SUC2 has a central role in the phloem loading of sucrose and is essential for high-performance sucrose transport from source to sink organs in *Arabidopsis* and presumably SUC2 homologs in most crop plants undertake similar key roles [2–4]. Recently, posttranscriptional modulation of SUC2 in coordination with a change in plant biomass has also been reported [18]. Also, transcriptional modulation of phloem-loading of SUC in sugar beet in response to exogenously fed sucrose has been investigated [19], however, the associated regulatory pathway remained unclear. In this context, signal cues and associated signaling components that directly regulate *SUC2* transcription and SUC2 activity have not been identified.

**Fig 7:**
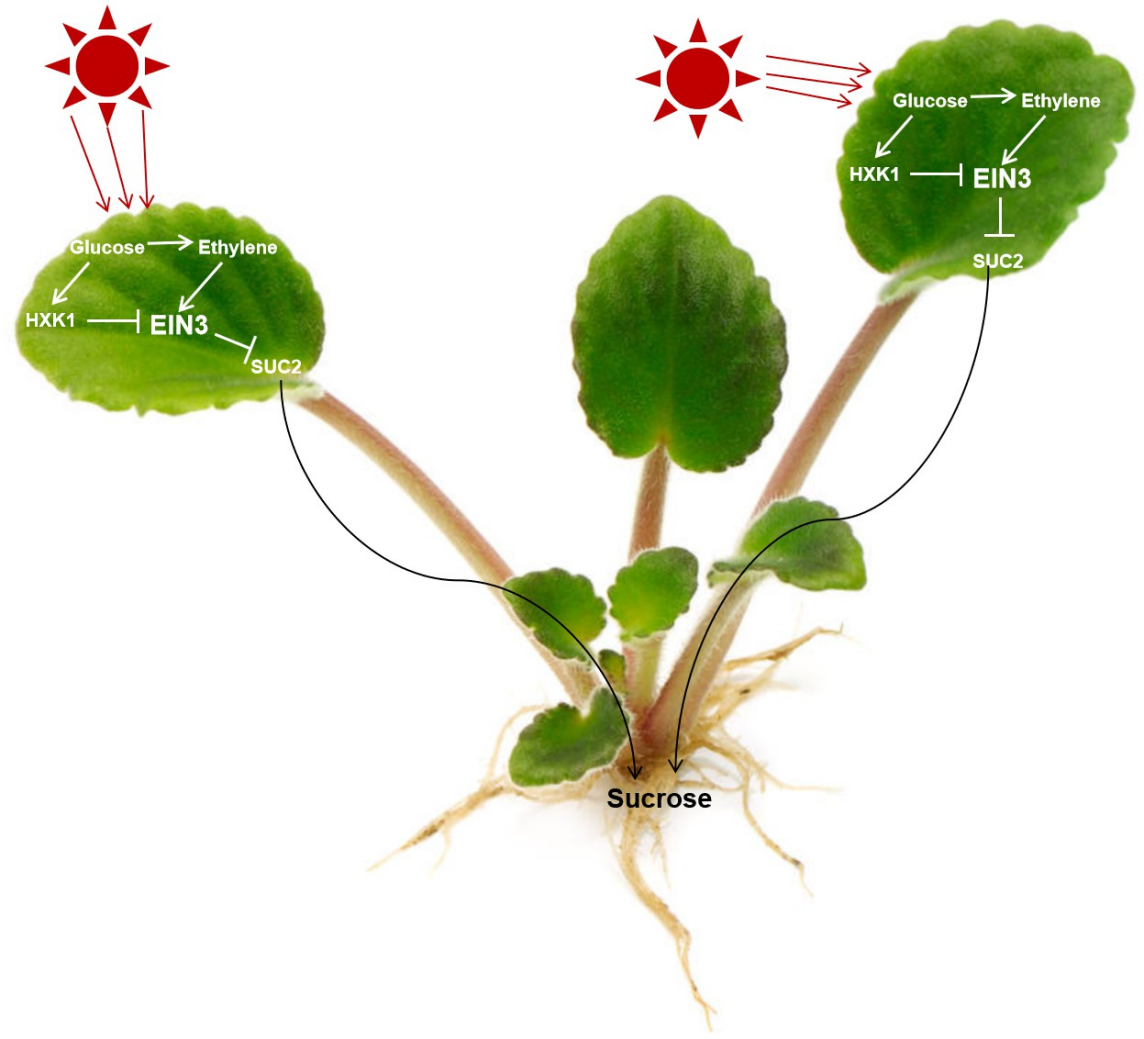
Model illustrating how glucose and ethylene signaling regulates sink root growth of *Arabidopsis* plants by controlling sucrose phloem loading. Sink root growth is promoted by the glucose signaling-HXK1-EIN3-SUC2 pathway, whereas sink root growth is inhibited by the ethylene signaling-EIN3-SUC2 pathway. The ethylene-EIN3 is ethylene canonical signal transduction pathway, which comprises the ethylene receptors ETR1, CTR1, EIN2, EIN3, and EIL1. The HXK1-EIN3 comprises that the regulation is via regulation of ubiquitination and proteolysis. Arrows and bars represent positive and negative regulation, respectively.

Our findings found that *EIN3*, acting the downstream of *HXK1*, functions upstream of *SUC2* in the modulation of glucose signaling mediated-root growth (Figs 1 and 5). Further, the transcription factor EIN3 directly binds to the *SUC2* promoter to inhibit *SUC2* expression (Fig 4) to suppress root growth (Figs 1 and 5). These components thus form the HXK1-**|**EIN3-**|**SUC2 module to promote sucrose phloem loading in source tissues and thereby raise sucrose levels in sink roots (Fig 6). As a result, glucose signaling mediated-root growth is facilitated (Figs 2 and 7). Further, EIN3 transcriptionally suppresses SUC2 activities, providing insight for a previous observation that down-regulation of sucrose phloem loading is linked to transcriptional regulation of SUC transporters [14]. Diverse environmental stresses also result in a change in *SUC* transcriptional levels [14]. However, increases in sucrose phloem loading may also result from post-transcriptional regulation [14], as a recent report has revealed posttranscriptional regulation of SUC2 in response to high-light acclimation [18].

In this study, HXK1 is central factor, and this protein has both signaling and enzymatic functions [7–8]. HXK1 is a key integration point of various plant hormone or external stimuli that modulate plant growth and metabolism [7–8]. Further, HXK1 signaling and enzymatic activities directly or indirectly affect many genes associated with photosynthesis, including key components of the photosynthetic machinery as well as RUBISCO and PAL [7]. Therefore, the HXK1-|EIN3-|SUC2 module may regulate the formation of major photosynthetic product sucrose, and sucrose transport from source leaves to sink roots (Fig 7). The resulting HXK1-|EIN3-|SUC2 module may promote sink root growth by the interaction of glucose and various plant hormone or external stimuli, as glucose and auxin signaling interaction control *Arabidopsis* sink root growth [5].

In the case of *Beta vulgaris* (Bv) SUC1 (a sugar beet leaf sucrose symporter), sucrose negatively and specifically regulates both sucrose transport activity and steady-state mRNA levels [19]. By contrast, in this work, with increasing age, sucrose/glucose accumulation was increased (Fig 6B and 6C), which in turn decreased EIN3 transcriptional activity (Fig 3C–3E) by decreasing EIN3 levels [9, 21] thereby raising *Arabidopsis thaliana* SUC2 (AtSUC2) dependent sucrose transport (Figs 6B and 6E and 7). Since *Solanum tuberosum* SUC4 (StSUC4) may play a role as a suppressor of StSUC1 [20], BvSUC1 and AtSUC2 may have opposite functions in the regulation of sucrose phloem loading.

However, when glucose accumulation in source leaves reaches a given threshold level of excess glucose, ethylene production is stimulated [15] and thus the level of EIN3 increases [9, 21], which in turn directly decreases SUC2 transport activity (Figs 4 and 7 and S6 Fig). Consequently, sink root growth is delayed, as ethylene inhibits root growth [22]. Therefore, we speculate that when glucose accumulation reaches a given threshold level, excess glucose stimulates ethylene production, which in turn inhibits root growth by its canonical signal transduction pathway suppressing SUC2 activity (Fig 7). Thus, excess levels of glucose consequently inhibit root growth via increased ethylene levels. Indeed, following a 3.5% glucose application, *ein3/eil1* plants had shortened root length (S7A and S7B Fig) and enhanced ethylene production (S7C Fig), relative to wild-type seedlings.

It has long been known that light promotes root growth. Light decreases EIN3 levels, whereas darkness elevates the accumulation of this transcriptional regulator [23]. When EIN3 levels are elevated [23] under darkness, *SUC2* transcripts are reduced [24]. Thus, darkness raises EIN3 protein levels and consequently decreases *SUC2* expression (Fig 3). As a result, this reduces sucrose phloem loading and thus enhances sucrose accumulation in source leaves. The resulting sucrose in sink roots is decreased, which in turn reduces root growth (S8 Fig).

Ethylene promotes hypocotyl growth [25], however, glucose and ethylene also have antagonistic functions in the regulation of plant growth [9]: hypocotyl length is decreased with an increasing sugar concentration [26]. Under basal conditions, HXK1 positively regulates hypocotyl growth [7]. However, under these conditions, EIN3 and SUC2 do not impact hypocotyl growth (S9 Fig). These findings suggest that the EIN3-SUC2 pathway may not be involved in the regulation of hypocotyl growth.

## Materials and methods

### Plant materials and growth conditions

The *5 × EBS-GUS*/wild-type [16], *ein3-1* and *ein3/eil1* [21] mutants, *35S*:*SUC2* and *suc2-5* [17] and *35S*:*EIN3* transgenic line [21] were all described previously.

The *ein3/suc2* mutant was obtained from F2 plants (*ein3-1/suc2-5*) that had elongated hypocotyls on solid MS medium with 6.0 μm ACC [21] and have increased anthocyanin accumulation in the petiole [17]. The *hxk1/ein3* mutant was obtained from F2 plants (*hxk1-3/ein3-1*) that had elongated hypocotyls on solid MS medium with 6.0 μm ACC [21] and have elongated primary roots grown on solid MS medium with 6% glucose [7]. *Agrobacterium tumefaciens* with *pCB2004EIN3pro*::*EIN3-GFP* were transformed into wild-type and *hxk1-3* plants. These plants were further characterized by using qPCR. Transgenic plants were generated using the *Agrobacterium tumefaciens*-mediated floral dip method [22].

For simultaneous germination, seeds were treated with vernalization of 4°C overnight and then sown on solid MS medium supplemented with 1% sucrose, pH 5.8 and 0.8% agar [22]. Seedlings grown on agar were maintained in a growth room under 16/8 h of light/dark cycle with cool white fluorescent light at 21 ± 2°C for long-day conditions [27]. Unless otherwise noted, plants grown in soil were maintained in a controlled environment growth chamber under short-day conditions (10h light/14 h dark) and normal light conditions (130 μmol quanta PAR m ^-2^ s ^-1^) or high light conditions (230 μmol quanta PAR m ^-2^ s ^-1^) at 22°C, as has been described by ref [28].

In all experiments, wild-type is the Col-0 background. Three biological replicates were performed with similar results, and error bars represent SD.

### Assay of sugar metabolites

Plant Sucrose and glucose Assay Kit (Beijing Solarbio Science & Technology Co., Ltd, Cat#BC2465; http://www.solarbio.com/goods-9298.html) was used for assay of sugar metabolites. 0.1g mature leaf blades were ground into homogenate at 23°C and then 0.5 ml extraction buffer added and centrifuged after extensive grinding and finally kept at 80°C for 10 min and shaken 3–5 times. After cooling these extracts were centrifuged at 4,000g for 10 min, the supernatant was transferred to a fresh tube and 2 mg reagent 5 added to decolorize at 80°C for 30 min. Then 0.5 ml extraction buffer was added, mixed and centrifuged at 4,000g for 10 min. The supernatant was transferred to a fresh tube for visible light analysis.

Three centrifugal tubes per sample were used with 25 μL of sample and then standard product (reagent 1) and water were added, respectively. Fifteen μL of reagent 2 was added, mixed and boiled at 100°C for 5 minutes. Subsequently, 175μL of reagent 3 and 50μL of reagent 4 were added, respectively. They were reacted in boiling water for 10 min, followed by measuring the light absorption value at 480 nm after cooling. The sucrose content was calculated accordingly, with respect to the sample standard absorption value and sample’s weight.

### Phloem loading of sucrose

Nine-day-old seedlings grown on solid MS medium with sugars or without were used to assay phloem loading of sucrose. Following incubation in pretreatment solution (pH5.7; liquid MS medium) for 40–50 mins, the cotyledons were incubated in the phloem loading buffer (pH 5.7; MS medium with 0.1–0.2% Sucrose) with [^14^ C] Sucrose (0.50μCi mL^-1^) and then were cultivated for 2–3 hours. Following three washes in desorption solution (pH 5.7; liquid MS medium and 1% Sucrose), these seedlings were put in scintillation vials and 3.0 mL of scintillation cocktail was added. The amount of [^14^ C] phloem loading of sucrose was analyzed on a scintillation counter and expressed as cpm mg^-1^ fresh weight.

### Confocal laser scanning microscope for GFP imaging

Subcellular localization of EIN3pro:EIN3-:Green Fluorescent Protein (GFP) fusion proteins was examined in the epidermis of 5-day-old transgenic cotyledons harbouring *EIN3pro*:*EIN3*:*GFP*.

These cotyledons were observed with a confocal laser scanning microscope (LSM 900; Zeiss) for GFP. All images were gained in the multitrack pattern and analyzed by using LSMimage-examiner software (Zeiss). The data were exported as eight-bit TIFF files and generated by using Adobe Photoshop 5.5 (Adobe Systems).

### Ethylene assay

These experiments were completed on 8-day-old wild-type and *ein3/eil1* plants by using a gas chromatograph equipped with a flame ionization detector [Echrom Technologies (Shanghai) Ltd Co], as was described [15].

### Plasmid constructs

For generating *pCB2004EIN3pro*::*EIN3-GFP* plasmids, the relevant primers are F-ggg gac aag ttt gta caa aaa agc agg ct AAT GTG TGG AAA CAT GGA TTT CT, R-ggg gac cac tttg tac aag aaa gct ggg t TTAGAACCATATGGATACAT; F-ggg gac aag ttt gta caa aaa agc agg ct cat att tgc atc tct cta tta gt; R-ggg gac cac tttg tac aag aaa gct ggg t atc gta ttt tta atc act cct aaa c were used.

### Quantitative PCR

Total RNA was extracted from tissues indicated in the figures by the TRIZOL reagent (Invitrogen), as has been described by refs [27–29]. SYBR green was used to monitor the kinetics of PCR product in real-time RT-PCR, as has been described by ref [27]. Primer pairs of *SUC2* (5′-ATC CAA TGG AGA AAG CTG CAA A-3′ and 5′-ACC ACA GAG CCA AAT CAG GAA-3′) were utilized. Primer pairs of *EIN3* have been described by ref [29].

### GUS assay and histochemical analysis of GUS activity

GUS assays were described previously [30–31]. In details, by using a mix buffer (0.4 mM of K3Fe(CN)6/K4Fe(CN)6, 1 mM X-gluc, 60 mM NaPO4 buffer, and 0.1% (v/v) Triton X-100), Transgenic plants were stained, followed by incubating at 37°C for 6–10 h. With GUS staining, chlorophyll was removed by using 30, 60, 70, 90 and 100% ethanol about 40 min for every process. Samples were observed by using stereoscopic microscope (SZX16, OLYMPUS). Histochemical analysis of GUS activity was undertaken as described previously [16]. In details, stained seedlings were transferred to microfuge tubes, which had a solution of 10 mM EDTA, 100 mM Na phosphate buffer, 2 mM potassium ferricyanide, 0.1% Triton X-100, pH 7.0, 1 mg/mL 5-bromo-4-chloro-3-indolyl-b- D -glucuronide and 2mM potassium ferrocyanide at 36–38°C over- night. These stained seedlings were cleared with an ethanol series.

### Transactivation assay

To examine EIN3 activity, we performed the below experiments. To generate *proSUC2-LUC*, the promoter was PCR amplified with primers *proSUC2-F* and *proSUC2-R* (*ProSUC2*-F-ttcctgcagcccgggggatcctaccagatttcggtaaattggtattcc; *ProSUC2*-R- tgtttttggcgtcttccatggatttgacaaaccaagaaagtaagaa) for the gnomic DNA of *Arabidopsis* and inserted into the cloning site of the *pGreen0800-LUC* vector.

The *MproSUC2-LUC* construct containing mutations in the S2 sequence of the *SUC2* promoter was generated using overlap extension PCR with primers (pGreen-SUC2pF: ttcctgcagcccgggggatcctaccagatttcggtaaattggtattcc, R-M-EMSA-SUC2: acacttatgaaatattgaaaCgCattaaataatCCCaaaatataaacataaatggtg, pGreen-SUC2pR: tgtttttggcgtcttccatggatttgacaaaccaagaaagtaagaa, M-EMSA-SUC2: caccatttatgtttatattttGGGattatttaatGcGtttcaatatttcataagtgt) and inserted into *pGreen0800-LUC* vector. Two fragments of *MproSUC2* were combined into a integrated fragments of *MproSUC2* by using primers (pGreen-SUC2pF: ttcctgcagcccgggggatcctaccagatttcggtaaattggtattcc, pGreen-SUC2pR: tgtttttggcgtcttccatggatttgacaaaccaagaaagtaagaa).

The *Agrobacterium* with the effector or reporter constructs were coincubated for 3 hour and then infiltrated into 20-day-old *N*. *Benthamiana* leaf blades. Leaf blades of these seedlings were incubated under middle light conditions (160 μmol quanta PAR m ^-2^ s ^-1^) for 2–4 days after infiltration. The firefly LUC activities were photographed after spraying with 1 mM luciferin. To determine the dualluciferase, activities of firefly luciferase and *Renilla* luciferase were measured, as has been previously described [32]. For LUC images, confocal laser scanning microscopy was performed by using a Epi-fluorescence microscopy (Olympus 80i, Olympus Corporation, Japan). The leaves were infiltrated with luciferin solution, and imaged by using a Tanon 5200S Luminescent Imaging Workstation.

### Protein expression and purification

The plasmid *pGEX-5X-1* for *EIN3* was utilzed. The coding sequence of *EIN3* was amplified by the primer pair (5′-GGATCC ATGATGTTTA ATGAGATGGG -3′ and 5′-CTCGAGTGCTCTGTTTGGGAT-3′) and cloned into the BamH1 and XhoI restriction sites of *pGEX-5X-1*. Recombinant glutathione S-transferase binding protein (GST)-tagged EIN3 was extracted from transformed *E*. *coli* (*Rosetta2*) after 10 h of incubation at 16°Cfollowing induction with 10 μM isopropylβ-D-1-thiogalactopyranoside. These recombinant proteins were purified using GST-agarose affinity.

### ChIP-PCR

The transgenic lines containing *35S*:*EIN3-GFP/ein3/eil1* were utilized. ChIP was performed with seedlings [28]. Leaf blades were incubated in buffer (1.0 mM PMSF, 0.5 M sucrose, 1mMEDTA, 10–12 mM Tris, pH8.0, and 1% formaldehyde) under vacuum for 15–20 min for crosslinking the chromatin. Then, 0.1 M Gly was placed in the mixture, incubating for an additional 5 min for terminating the reaction. Leaf blades were placed and ground in liquid nitrogen and re-suspended to lysis buffer (150mMNaCl,1mMEDTA, 0.1% SDS, 0.1%deoxycholate, 50 mM HEPES, pH7.5, 1%TritonX-100, 10 mM Na-butyrate, 1 mM PMSF, and 13 complete protease inhibitor [Roche]). Chromatin was sheared to about 200 to 500 bp fragments via sonication followed by centrifuged. At 4°C, supernatants were precleared under protein G agarose beads for 1–1.5h. Input material (supernatant containing chromatin) was used for immunoprecipitation with anti-GFP antibody. Anti-GFP antibody bound to EIN3-FLAG or GFP-chromatin complexes was incubated with protein G agarose beads for 1–1.5 h at 4–6°C and then washed several times and eluted with elution buffer. Input and immunoprecipitated chromatin were uncross-linked for 6h at 6°C with 5M NaCl. Immunoprecipitated chromatin and input were used for PCR analysis The ChIP DNA products were analyzed PCR using two pairs of primers that were synthesized to amplify ~200 bp DNA fragments in the promoter region of *SUC2*. Primer sequences (CHIP-SUC2-1F: atgcatgcaaaatagcacaccatttatgt, CHIP-SUC2-1R: actgcattttcacctctcccactgt; CHIP-SUC1-1F: Acagtgggagaggtgaaaatgcag CHIP-SUC1-1R: gcgagtggcactagaataattttcggtgat; SUC2 3F: GTTTTTCTCGTTTTTCATGGCG SUC2 3R: ACTATGAGGAGAAGCGTTATGG; CHIP-SUC2-4F: actttgcttatgtgattgcctgaggat CHIP-SUC2-4R: tgctattttgcatgcatataatcacctttttga) were used.

### Electrophoretic Mobility Shift Assay (EMSA)

The electrophoresis mobility shift assay (EMSA) was performed by using the LightShift Chemiluminescent EMSA Kit (Pierce, 20148) according to the manufacturer’ instructions. The biotin-labeled *SUC2*-*S2* DNA fragments and its mutated derivatives (EMSA-SUC2: caccatttatgtttatattttcaaattatttaatacatttcaatatttcataagtgt R-EMSA-SUC2: S-acacttatgaaatattgaaatgtattaaataatttgaaaatataaacataaatggtg biotin-EMSA-SUC2: Caccatttatgtttatattttcaaattatttaatacatttcaatatttcataagtgt R-biotin-EMSA-SUC2: acacttatgaaatattgaaatgtattaaataatttgaaaatataaacataaatggtg; M-EMSA-SUC2: caccatttatgtttatattttGGGattatttaatGcGtttcaatatttcataagtgt R-M-EMSA-SUC2: acacttatgaaatattgaaaCgCattaaataatCCCaaaatataaacataaatggtg) were synthesized, annealed and used as probes. The corresponding biotin unlabeled DNA fragments were utilized as competitor sequences in the assay. The probes were incubated with EIN3 fusion protein at room temperature for 20 mins in a binding buffer (56 concentrations: 50 mM HEPES-KOH [pH 7.5], 375 mM KCl, 6.25 mM MgCl, 1 mM DTT, 0.5mg/mL BSA, Glycerol 25%). Each 20 ml binding reaction contained 25 fmol Biotin-probe, 6mg protein, and 1mg Poly (dIdC) to minimize nonspecific interactions. The reaction products were analyzed by 6.5% native polyacrylamide gel electrophoresis. This experimental process has been described previously [30,33].

## Supporting information

S1 Fig The *gin2-1* mutant had shortened primary roots. (PPTX)

S2 Fig *35S*:*EIN3* and *35S*:*SUC2* seedlings were identified by using qPCR. (PPTX)

S3 Fig Primary root length of *35S*:*EIN3*, *eil1-3*, *ein3/eil1*, wild-type (Col-0), and *ein3-1* seedlings under no sugar conditions. (PPTX)

S4 Fig *EIN3/EIL1* negatively regulates primary root growth. (PPTX)

S5 Fig *SUC2* positively regulates primary root growth. (PPTX)

S6 Fig Sucrose phloem loading of *35S*:*SUC2* mature leaves with ACC treatment. (PPTX)

S7 Fig The *ein3/eil1* root growth under excess glucose levels. (PPTX)

S8 Fig The *ein3/eil1* mutant has shortened primary root length in the dark. (PPTX)

S9 Fig The HXK1-EIN3-SUC2 pathway may be involved in the regulation of hypocotyl growth. (PPTX)
