# Supplementary material for: Long-distance transport of sucrose in source leaves promotes sink root growth by the EIN3-SUC2 module
Source: PLoS Genet. 2022 Sep 21;18(9):e1010424. doi: 10.1371/journal.pgen.1010424 (PMC9529141; doi:10.1371/journal.pgen.1010424)
Supplement: S1 Fig — (PPTX) [file pgen.1010424.s001.pptx]

## Slide 1
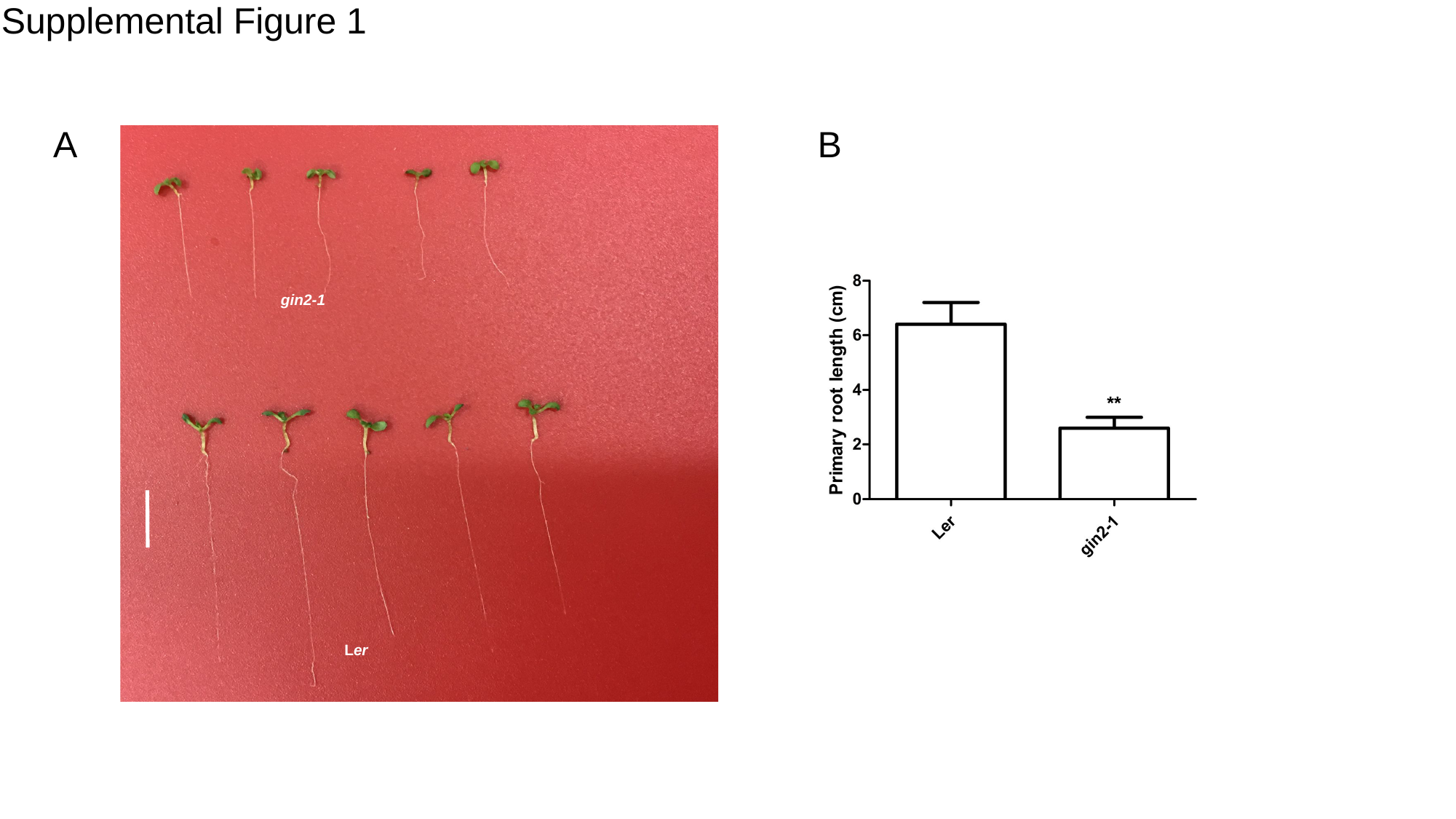

Supplemental Figure 1
A
B
gin2-1
**
Ler

## Slide 2
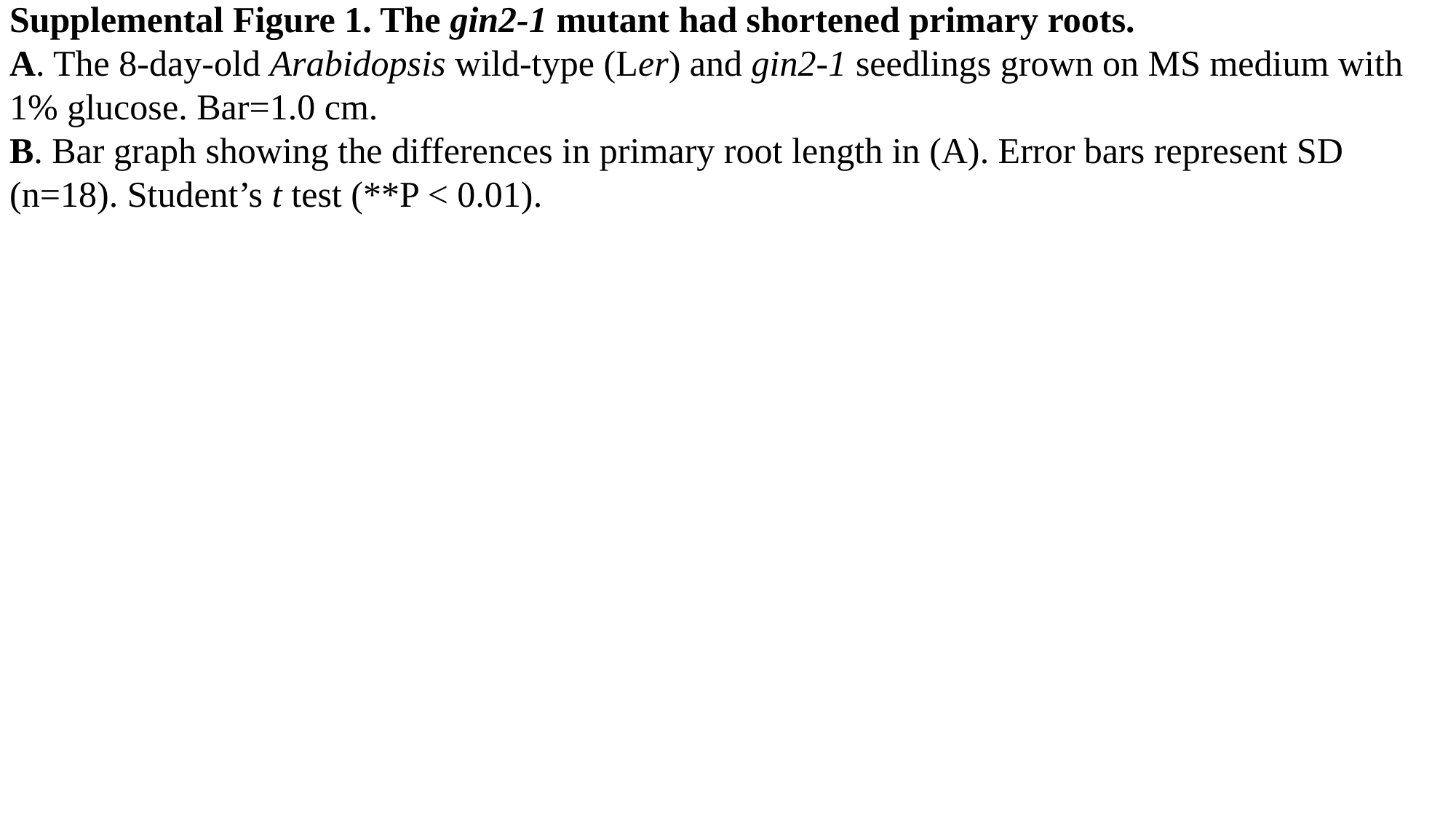

Supplemental Figure 1. The gin2-1 mutant had shortened primary roots.
A. The 8-day-old Arabidopsis wild-type (Ler) and gin2-1 seedlings grown on MS medium with 1% glucose. Bar=1.0 cm.
B. Bar graph showing the differences in primary root length in (A). Error bars represent SD (n=18). Student’s t test (**P < 0.01).
