# Supplementary material for: Long-distance transport of sucrose in source leaves promotes sink root growth by the EIN3-SUC2 module
Source: PLoS Genet. 2022 Sep 21;18(9):e1010424. doi: 10.1371/journal.pgen.1010424 (PMC9529141; doi:10.1371/journal.pgen.1010424)
Supplement: S2 Fig — (PPTX) [file pgen.1010424.s002.pptx]

## Slide 1
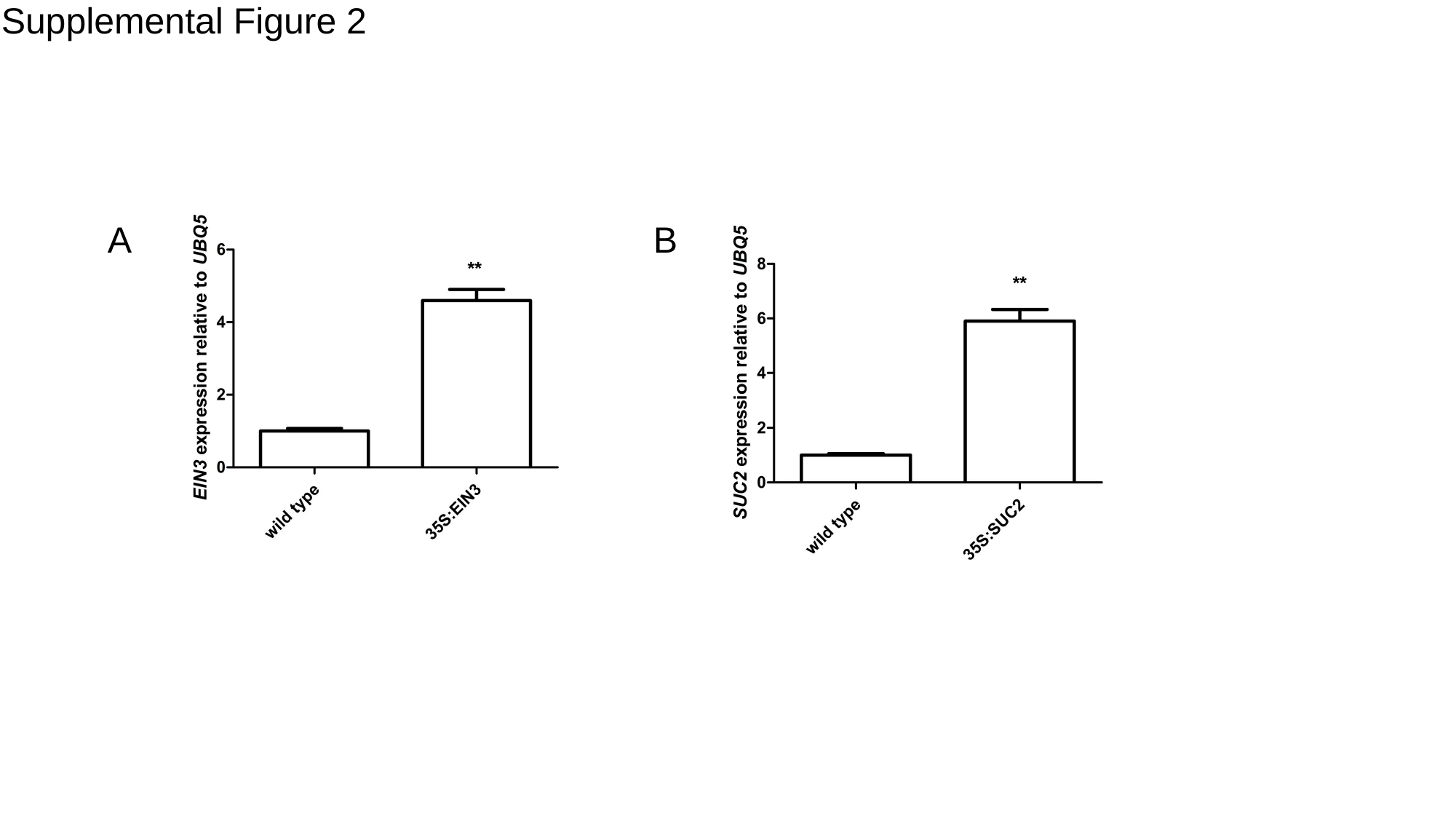

Supplemental Figure 2
A
B
**
**

## Slide 2
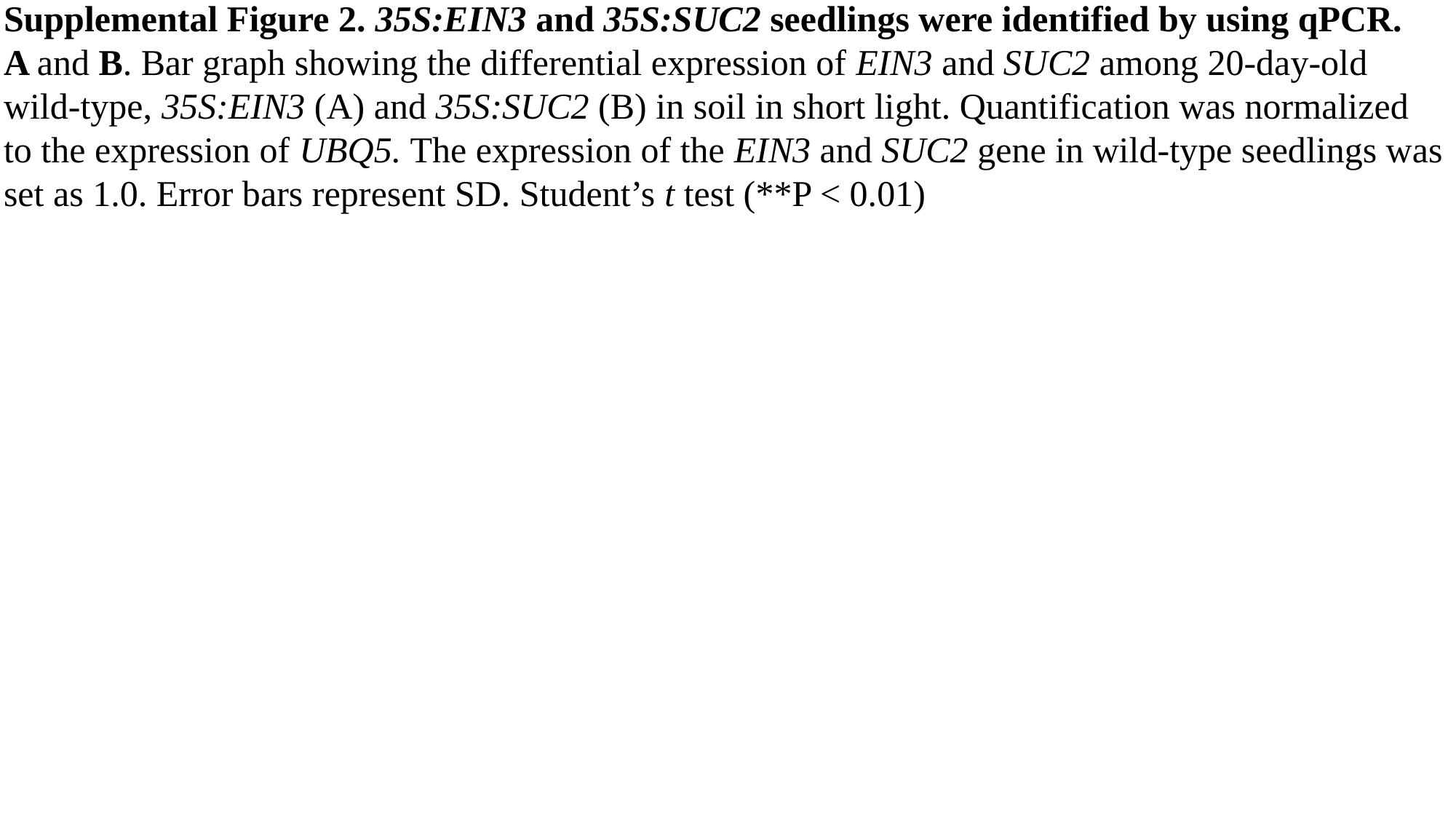

Supplemental Figure 2. 35S:EIN3 and 35S:SUC2 seedlings were identified by using qPCR.
A and B. Bar graph showing the differential expression of EIN3 and SUC2 among 20-day-old wild-type, 35S:EIN3 (A) and 35S:SUC2 (B) in soil in short light. Quantification was normalized to the expression of UBQ5. The expression of the EIN3 and SUC2 gene in wild-type seedlings was set as 1.0. Error bars represent SD. Student’s t test (**P < 0.01)
