# Supplementary material for: Long-distance transport of sucrose in source leaves promotes sink root growth by the EIN3-SUC2 module
Source: PLoS Genet. 2022 Sep 21;18(9):e1010424. doi: 10.1371/journal.pgen.1010424 (PMC9529141; doi:10.1371/journal.pgen.1010424)
Supplement: S3 Fig — (PPTX) [file pgen.1010424.s003.pptx]

## Slide 1
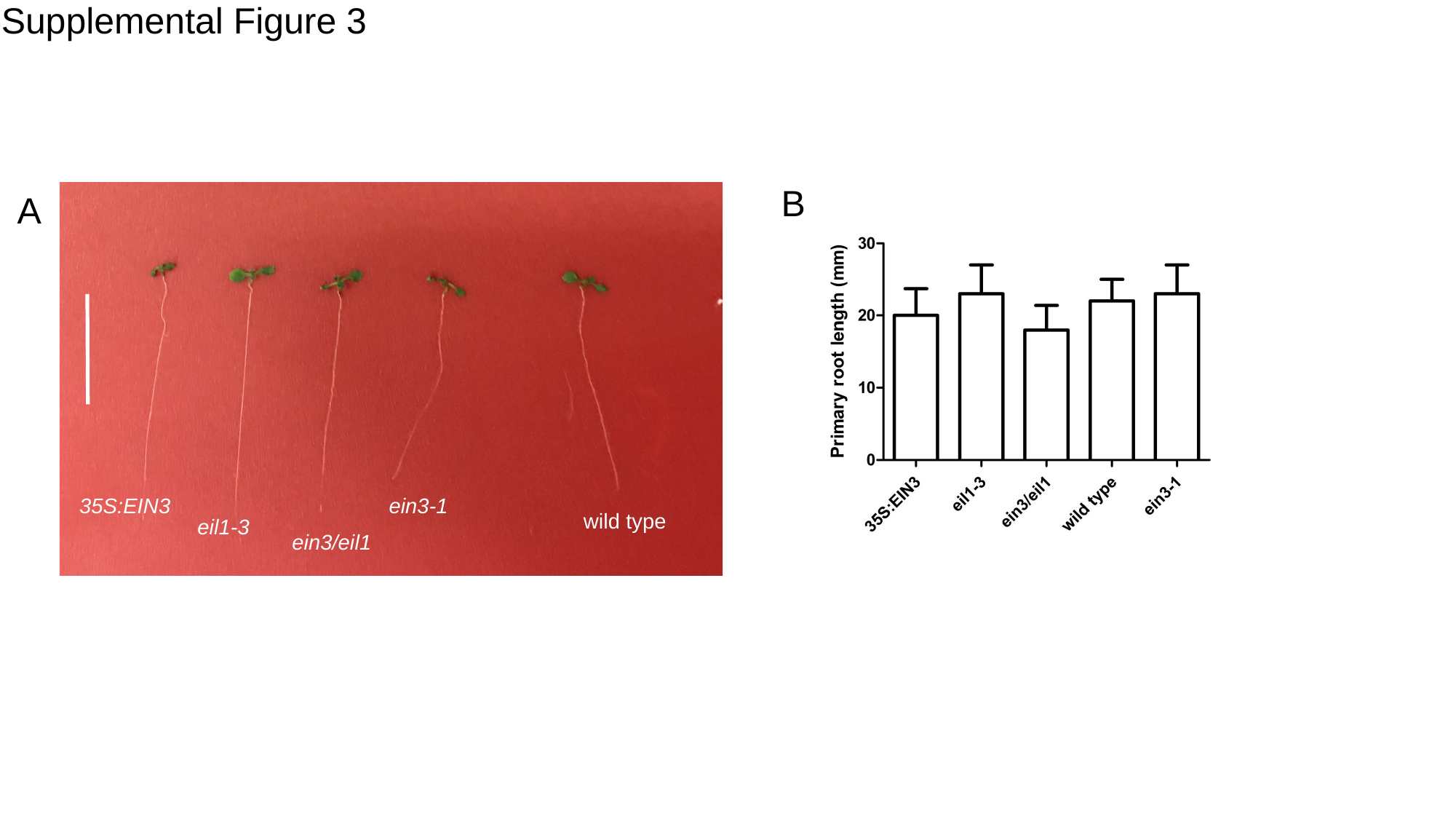

Supplemental Figure 3
B
A
35S:EIN3
ein3-1
wild type
eil1-3
ein3/eil1

## Slide 2
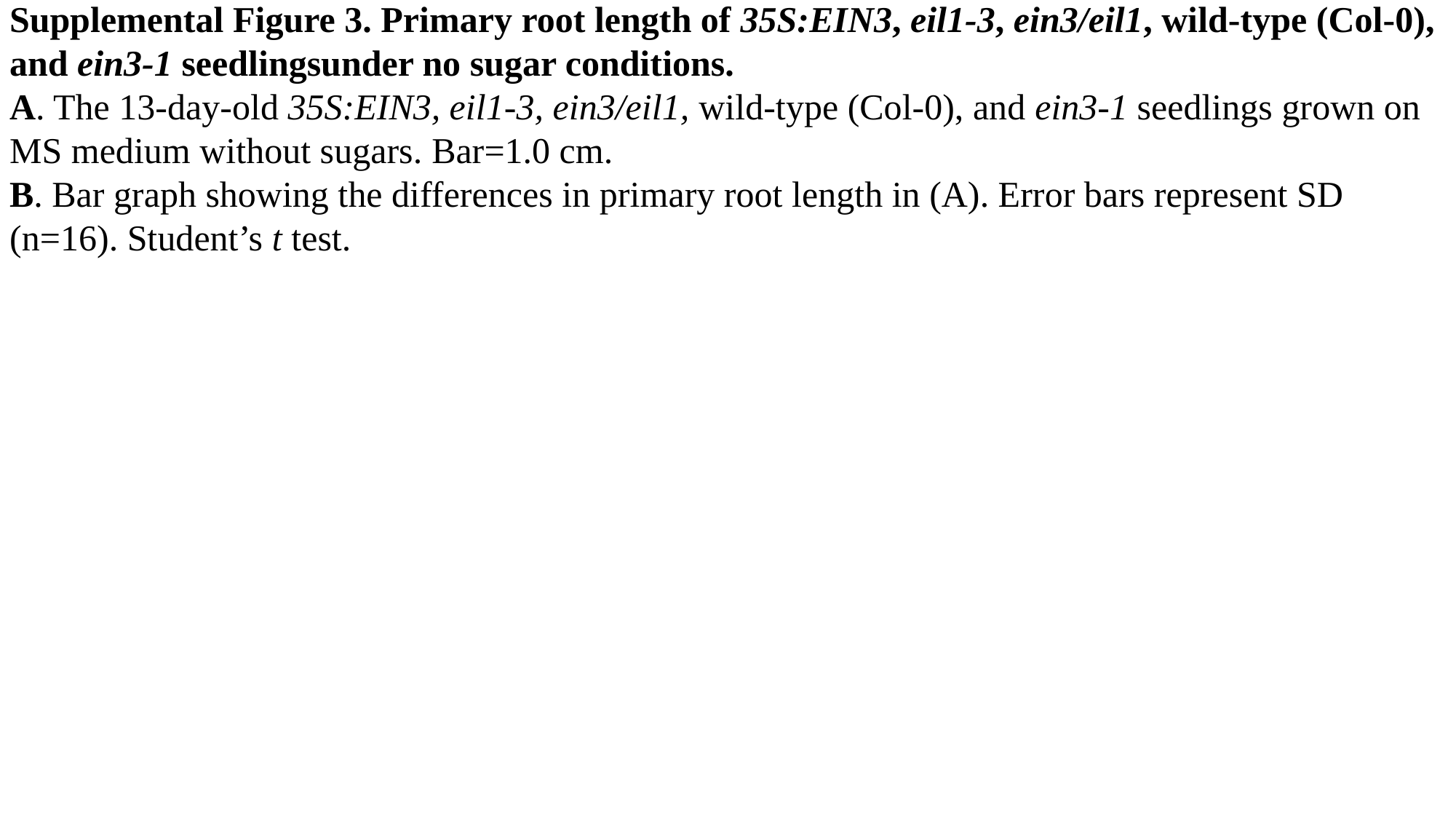

Supplemental Figure 3. Primary root length of 35S:EIN3, eil1-3, ein3/eil1, wild-type (Col-0), and ein3-1 seedlingsunder no sugar conditions.
A. The 13-day-old 35S:EIN3, eil1-3, ein3/eil1, wild-type (Col-0), and ein3-1 seedlings grown on MS medium without sugars. Bar=1.0 cm.
B. Bar graph showing the differences in primary root length in (A). Error bars represent SD (n=16). Student’s t test.
