# Supplementary material for: Long-distance transport of sucrose in source leaves promotes sink root growth by the EIN3-SUC2 module
Source: PLoS Genet. 2022 Sep 21;18(9):e1010424. doi: 10.1371/journal.pgen.1010424 (PMC9529141; doi:10.1371/journal.pgen.1010424)
Supplement: S4 Fig — (PPTX) [file pgen.1010424.s004.pptx]

## Slide 1
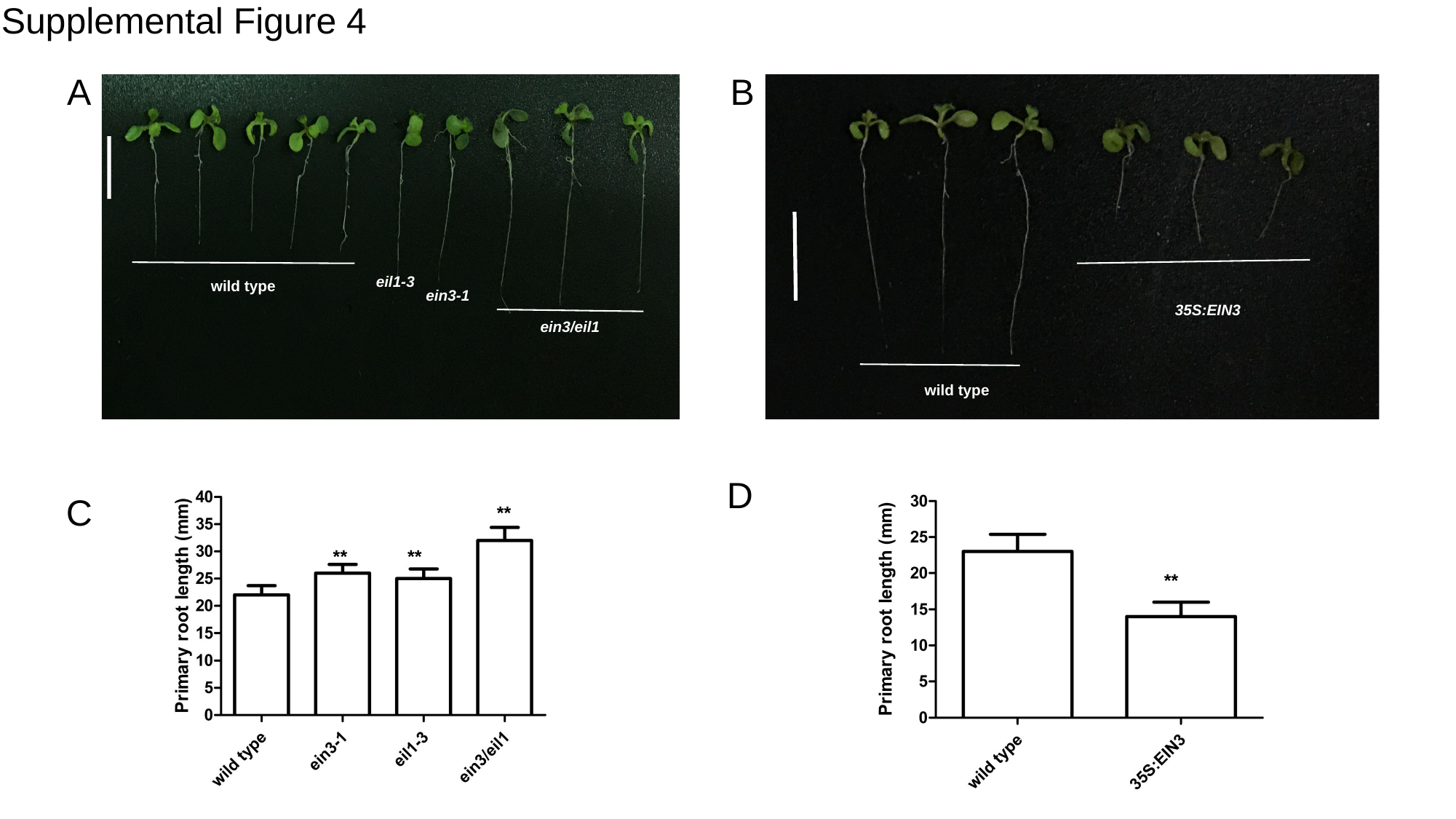

Supplemental Figure 4
A
B
eil1-3
wild type
ein3-1
35S:EIN3
ein3/eil1
wild type
D
C
**
**
**
**

## Slide 2
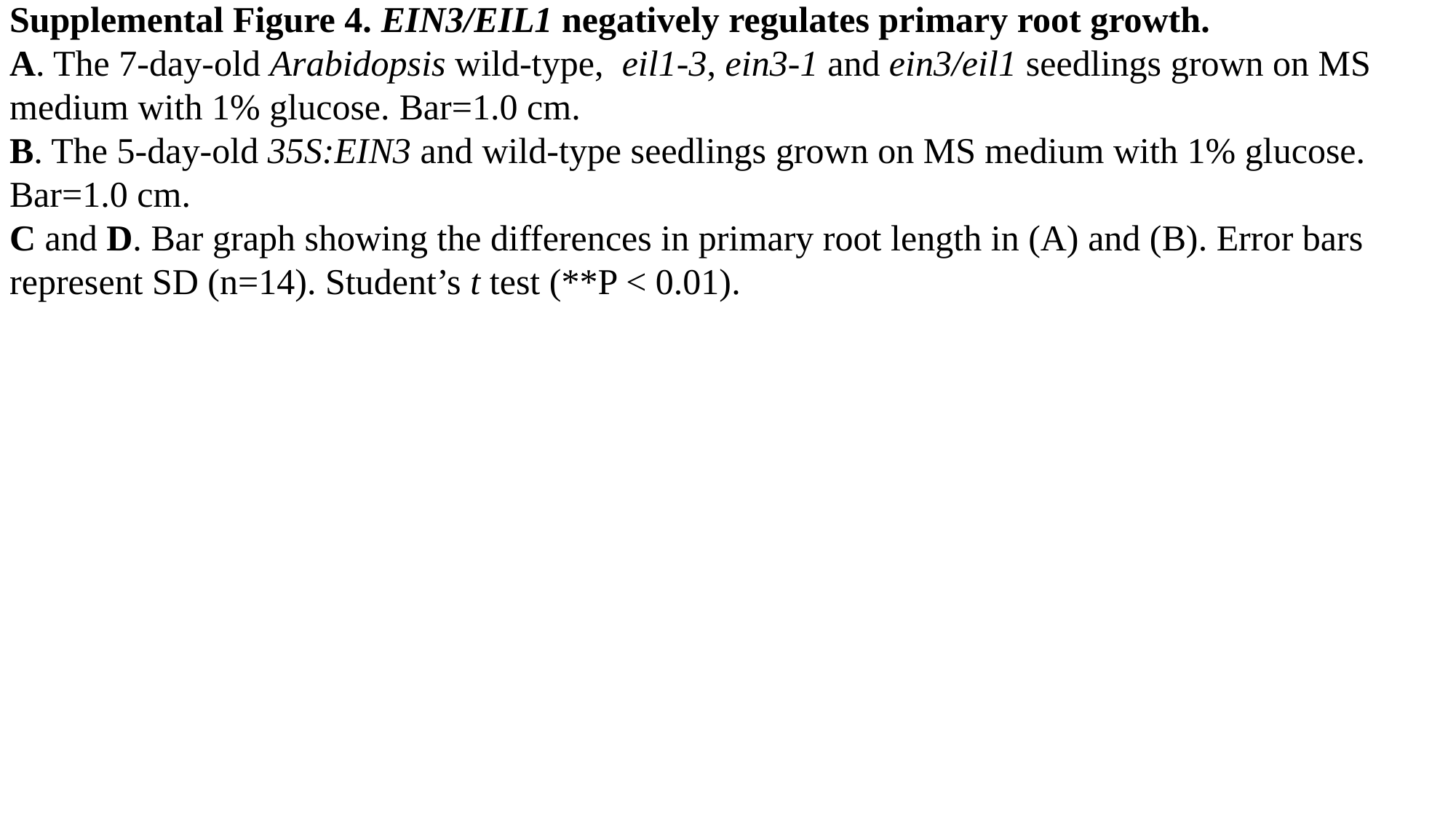

Supplemental Figure 4. EIN3/EIL1 negatively regulates primary root growth.
A. The 7-day-old Arabidopsis wild-type, eil1-3, ein3-1 and ein3/eil1 seedlings grown on MS medium with 1% glucose. Bar=1.0 cm.
B. The 5-day-old 35S:EIN3 and wild-type seedlings grown on MS medium with 1% glucose. Bar=1.0 cm.
C and D. Bar graph showing the differences in primary root length in (A) and (B). Error bars represent SD (n=14). Student’s t test (**P < 0.01).
