# Supplementary material for: Long-distance transport of sucrose in source leaves promotes sink root growth by the EIN3-SUC2 module
Source: PLoS Genet. 2022 Sep 21;18(9):e1010424. doi: 10.1371/journal.pgen.1010424 (PMC9529141; doi:10.1371/journal.pgen.1010424)
Supplement: S5 Fig — (PPTX) [file pgen.1010424.s005.pptx]

## Slide 1
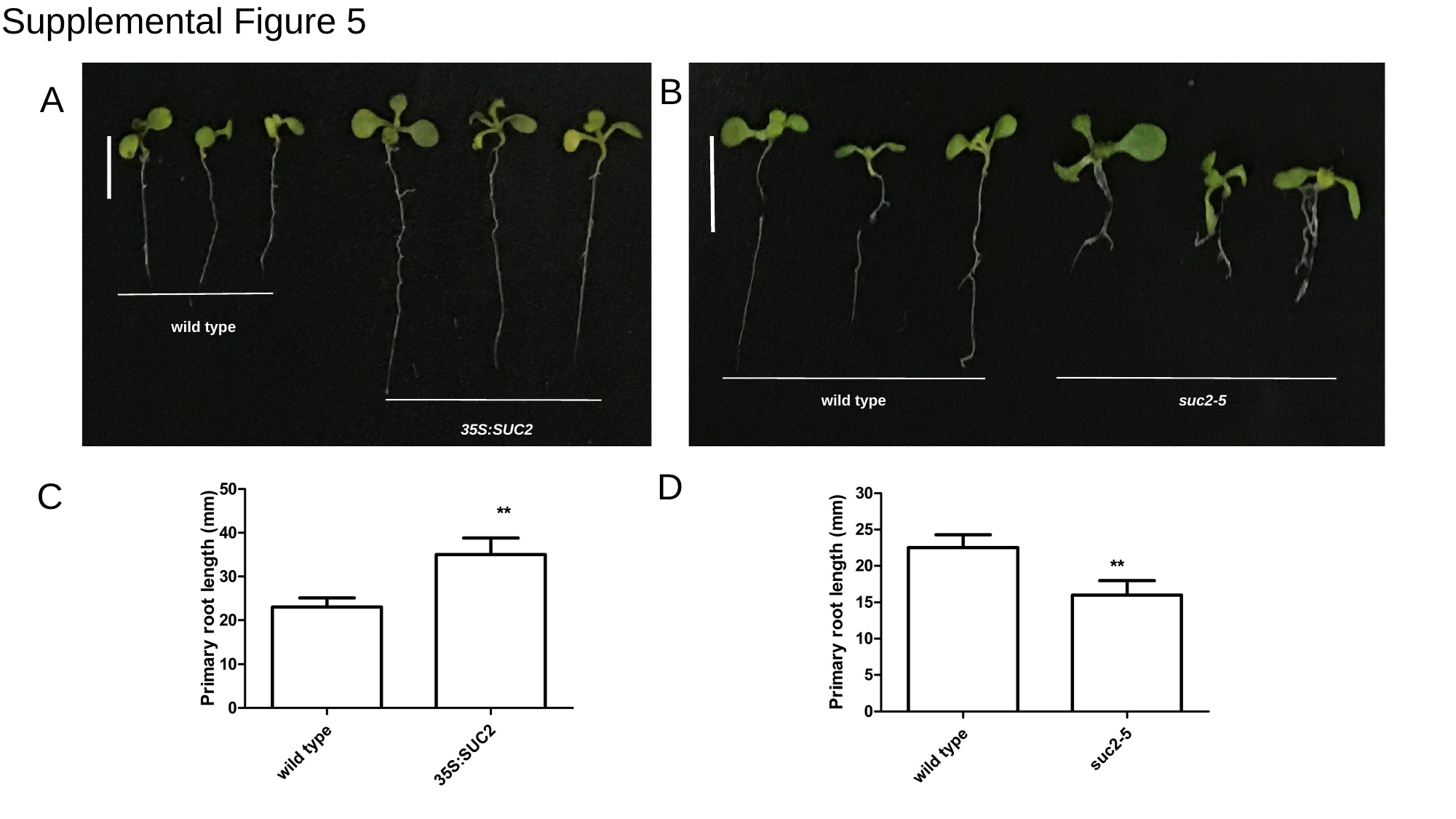

Supplemental Figure 5
B
A
wild type
wild type
suc2-5
35S:SUC2
D
C
**
**

## Slide 2
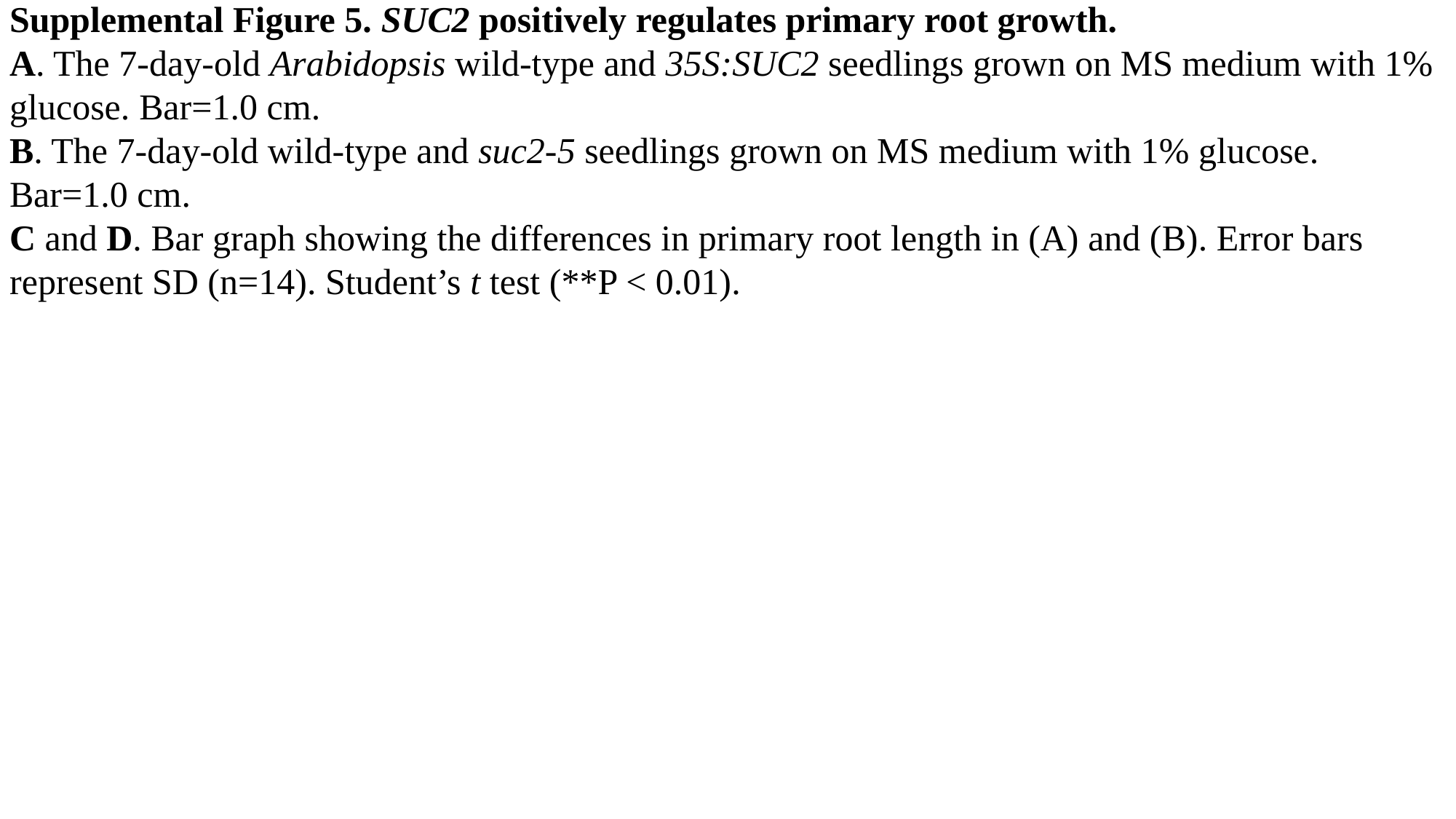

Supplemental Figure 5. SUC2 positively regulates primary root growth.
A. The 7-day-old Arabidopsis wild-type and 35S:SUC2 seedlings grown on MS medium with 1% glucose. Bar=1.0 cm.
B. The 7-day-old wild-type and suc2-5 seedlings grown on MS medium with 1% glucose. Bar=1.0 cm.
C and D. Bar graph showing the differences in primary root length in (A) and (B). Error bars represent SD (n=14). Student’s t test (**P < 0.01).
