# Supplementary material for: Long-distance transport of sucrose in source leaves promotes sink root growth by the EIN3-SUC2 module
Source: PLoS Genet. 2022 Sep 21;18(9):e1010424. doi: 10.1371/journal.pgen.1010424 (PMC9529141; doi:10.1371/journal.pgen.1010424)
Supplement: S6 Fig — (PPTX) [file pgen.1010424.s006.pptx]

## Slide 1
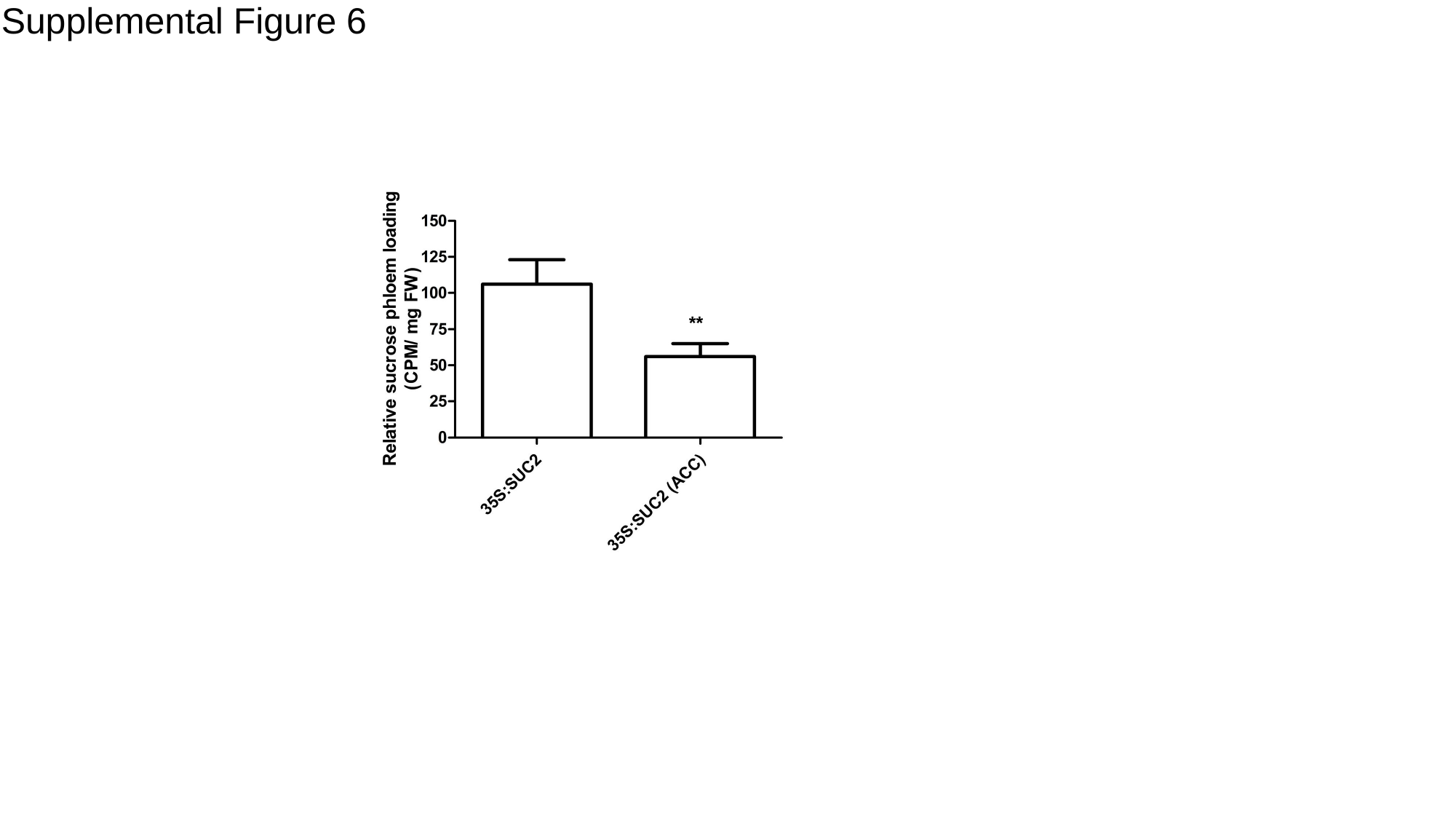

Supplemental Figure 6
**

## Slide 2
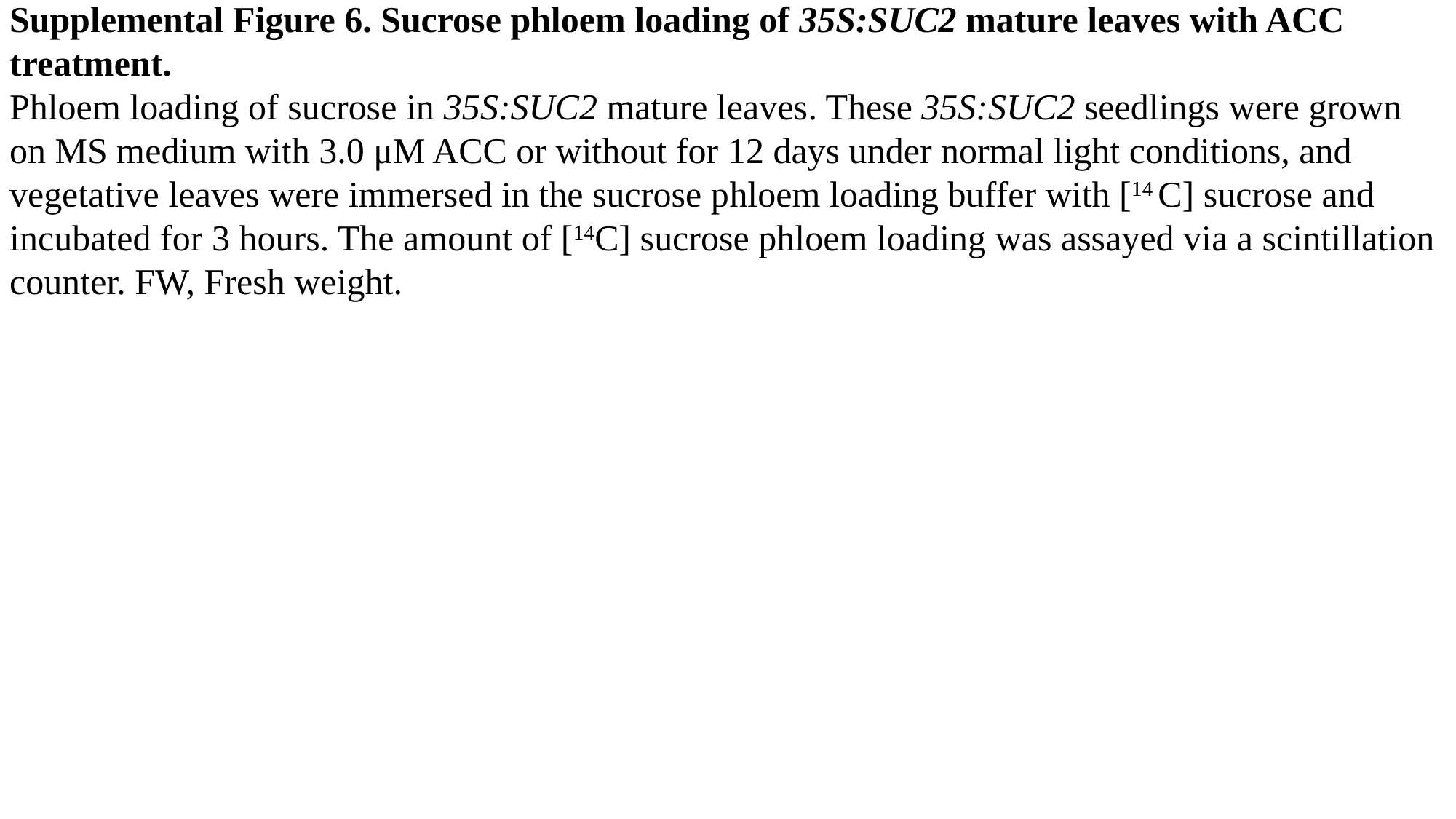

Supplemental Figure 6. Sucrose phloem loading of 35S:SUC2 mature leaves with ACC treatment.
Phloem loading of sucrose in 35S:SUC2 mature leaves. These 35S:SUC2 seedlings were grown on MS medium with 3.0 μM ACC or without for 12 days under normal light conditions, and vegetative leaves were immersed in the sucrose phloem loading buffer with [14 C] sucrose and incubated for 3 hours. The amount of [14C] sucrose phloem loading was assayed via a scintillation counter. FW, Fresh weight.
