# Supplementary material for: Long-distance transport of sucrose in source leaves promotes sink root growth by the EIN3-SUC2 module
Source: PLoS Genet. 2022 Sep 21;18(9):e1010424. doi: 10.1371/journal.pgen.1010424 (PMC9529141; doi:10.1371/journal.pgen.1010424)
Supplement: S7 Fig — (PPTX) [file pgen.1010424.s007.pptx]

## Slide 1
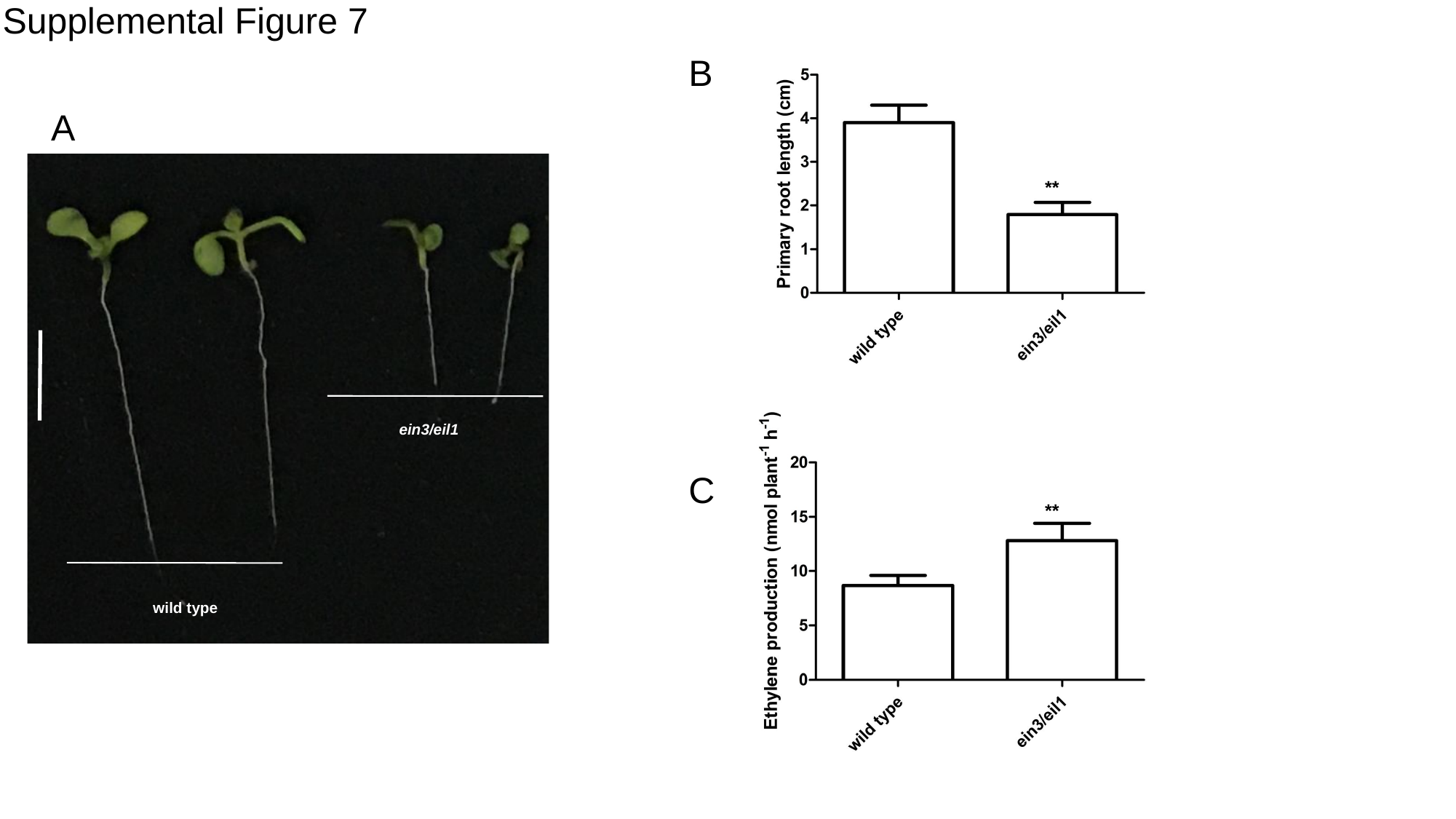

Supplemental Figure 7
B
A
**
ein3/eil1
C
**
wild type

## Slide 2
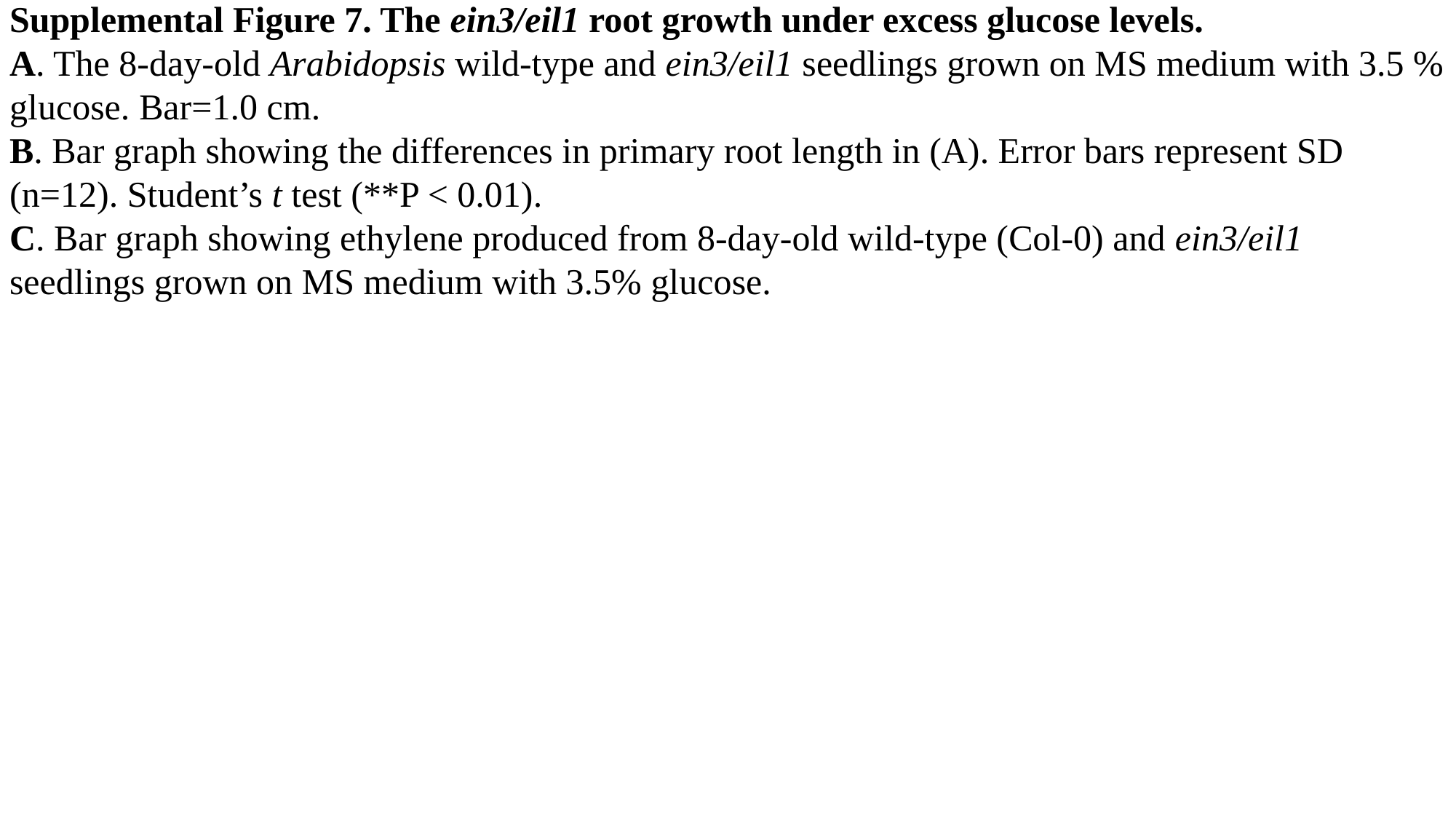

Supplemental Figure 7. The ein3/eil1 root growth under excess glucose levels.
A. The 8-day-old Arabidopsis wild-type and ein3/eil1 seedlings grown on MS medium with 3.5 % glucose. Bar=1.0 cm.
B. Bar graph showing the differences in primary root length in (A). Error bars represent SD (n=12). Student’s t test (**P < 0.01).
C. Bar graph showing ethylene produced from 8-day-old wild-type (Col-0) and ein3/eil1 seedlings grown on MS medium with 3.5% glucose.
