# Supplementary material for: Long-distance transport of sucrose in source leaves promotes sink root growth by the EIN3-SUC2 module
Source: PLoS Genet. 2022 Sep 21;18(9):e1010424. doi: 10.1371/journal.pgen.1010424 (PMC9529141; doi:10.1371/journal.pgen.1010424)
Supplement: S8 Fig — (PPTX) [file pgen.1010424.s008.pptx]

## Slide 1
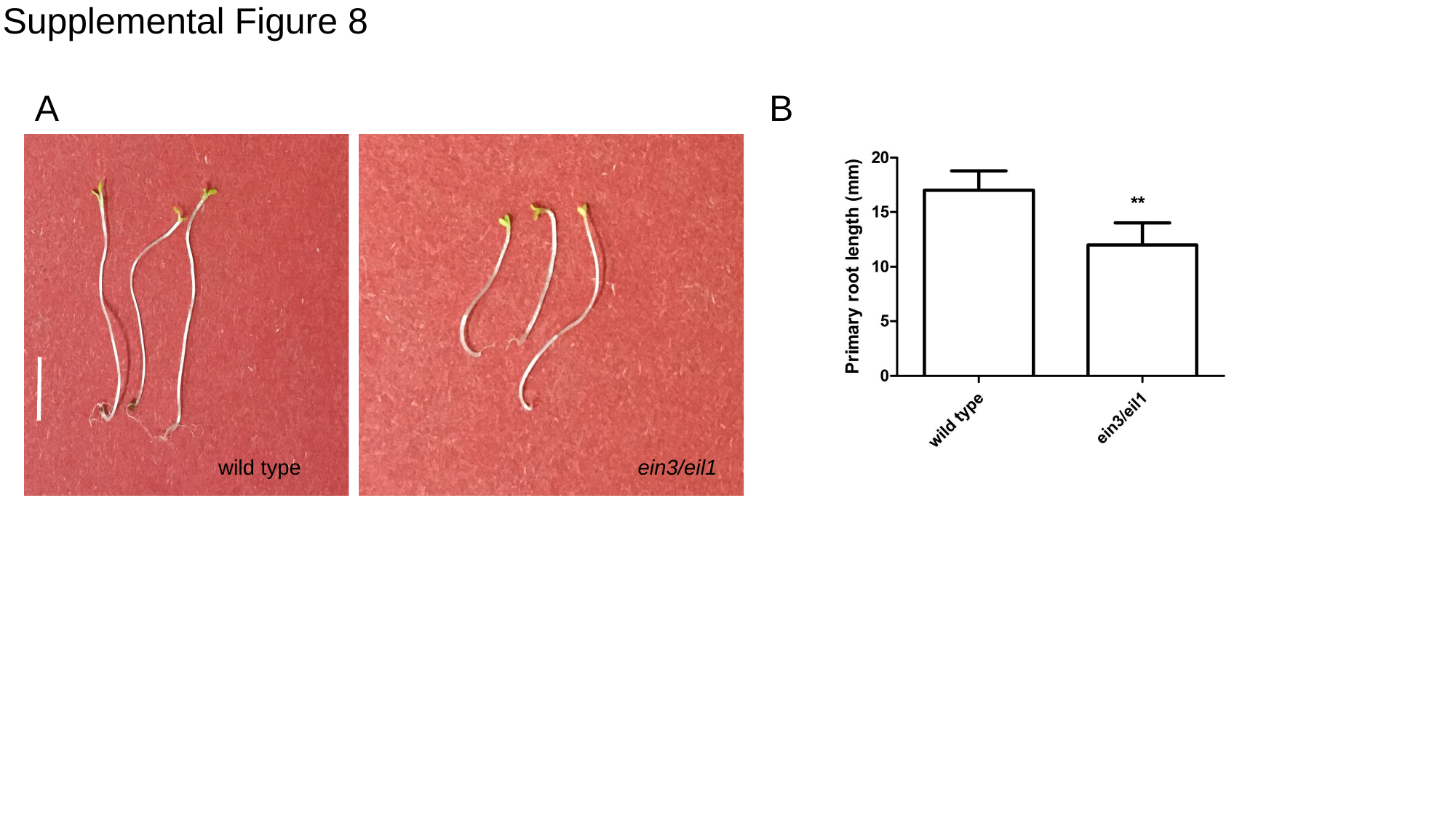

Supplemental Figure 8
A
B
**
wild type
ein3/eil1

## Slide 2
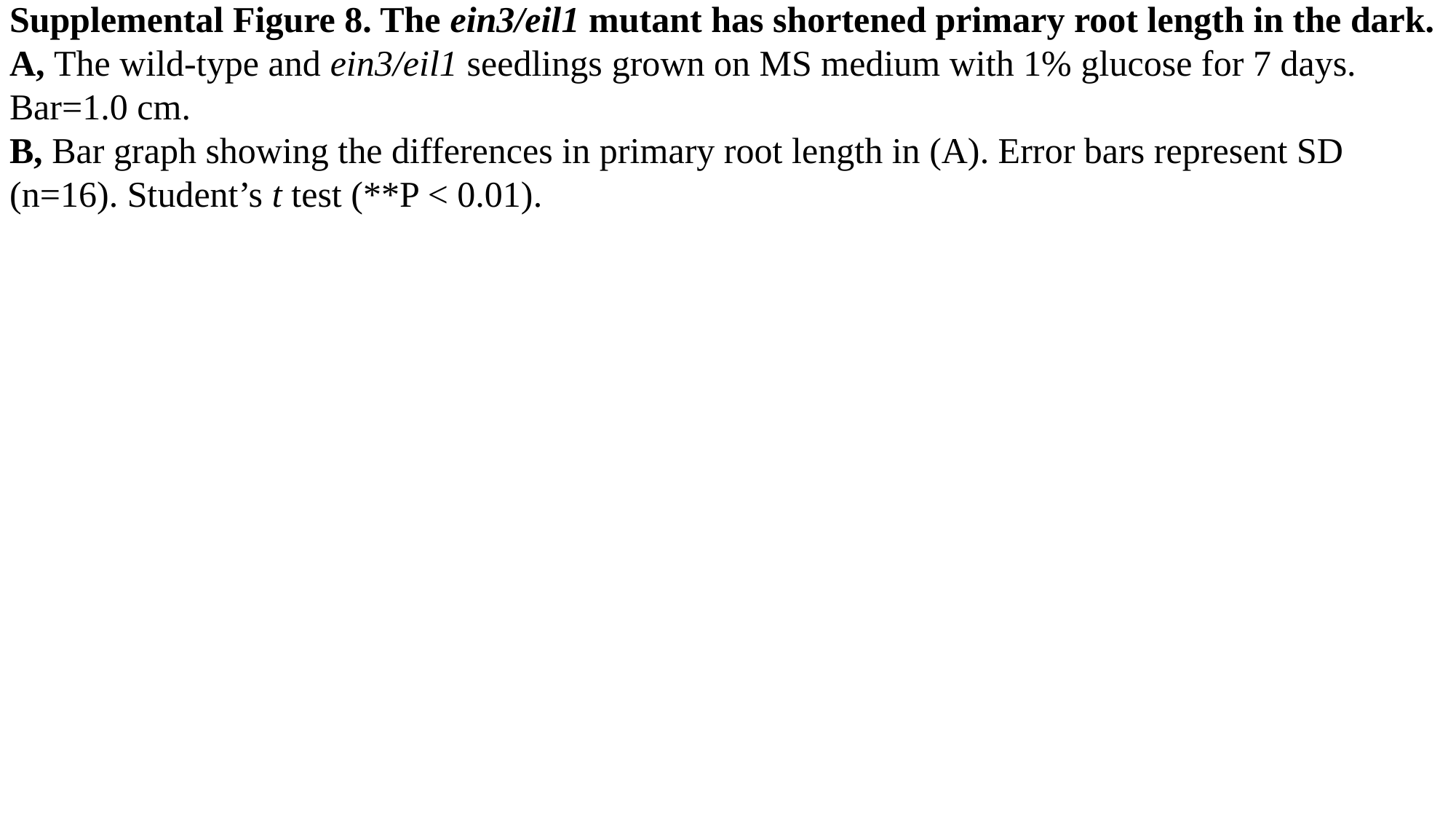

Supplemental Figure 8. The ein3/eil1 mutant has shortened primary root length in the dark.
A, The wild-type and ein3/eil1 seedlings grown on MS medium with 1% glucose for 7 days. Bar=1.0 cm.
B, Bar graph showing the differences in primary root length in (A). Error bars represent SD (n=16). Student’s t test (**P < 0.01).
