# Supplementary material for: Long-distance transport of sucrose in source leaves promotes sink root growth by the EIN3-SUC2 module
Source: PLoS Genet. 2022 Sep 21;18(9):e1010424. doi: 10.1371/journal.pgen.1010424 (PMC9529141; doi:10.1371/journal.pgen.1010424)
Supplement: S9 Fig — (PPTX) [file pgen.1010424.s009.pptx]

## Slide 1
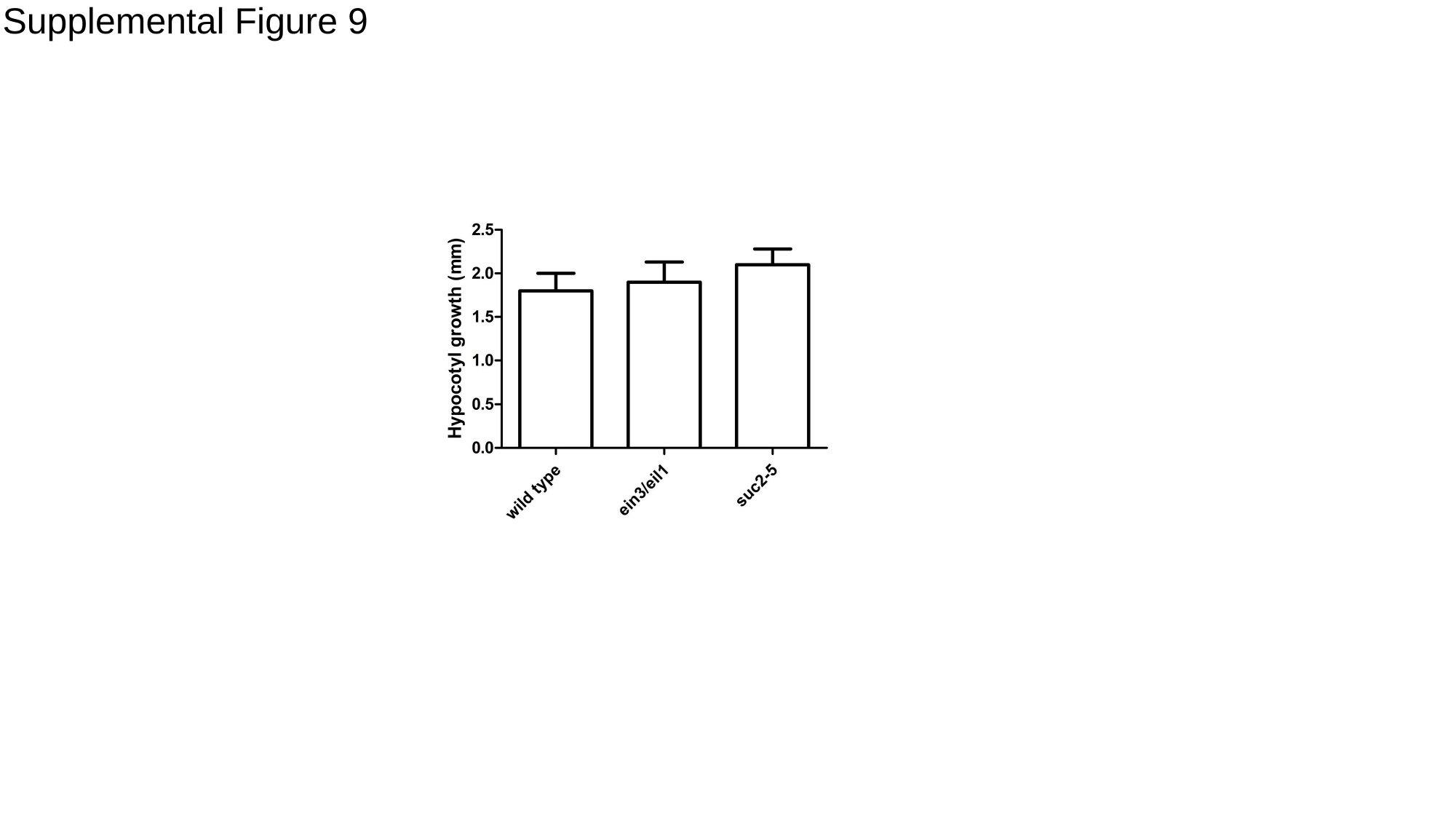

Supplemental Figure 9

## Slide 2
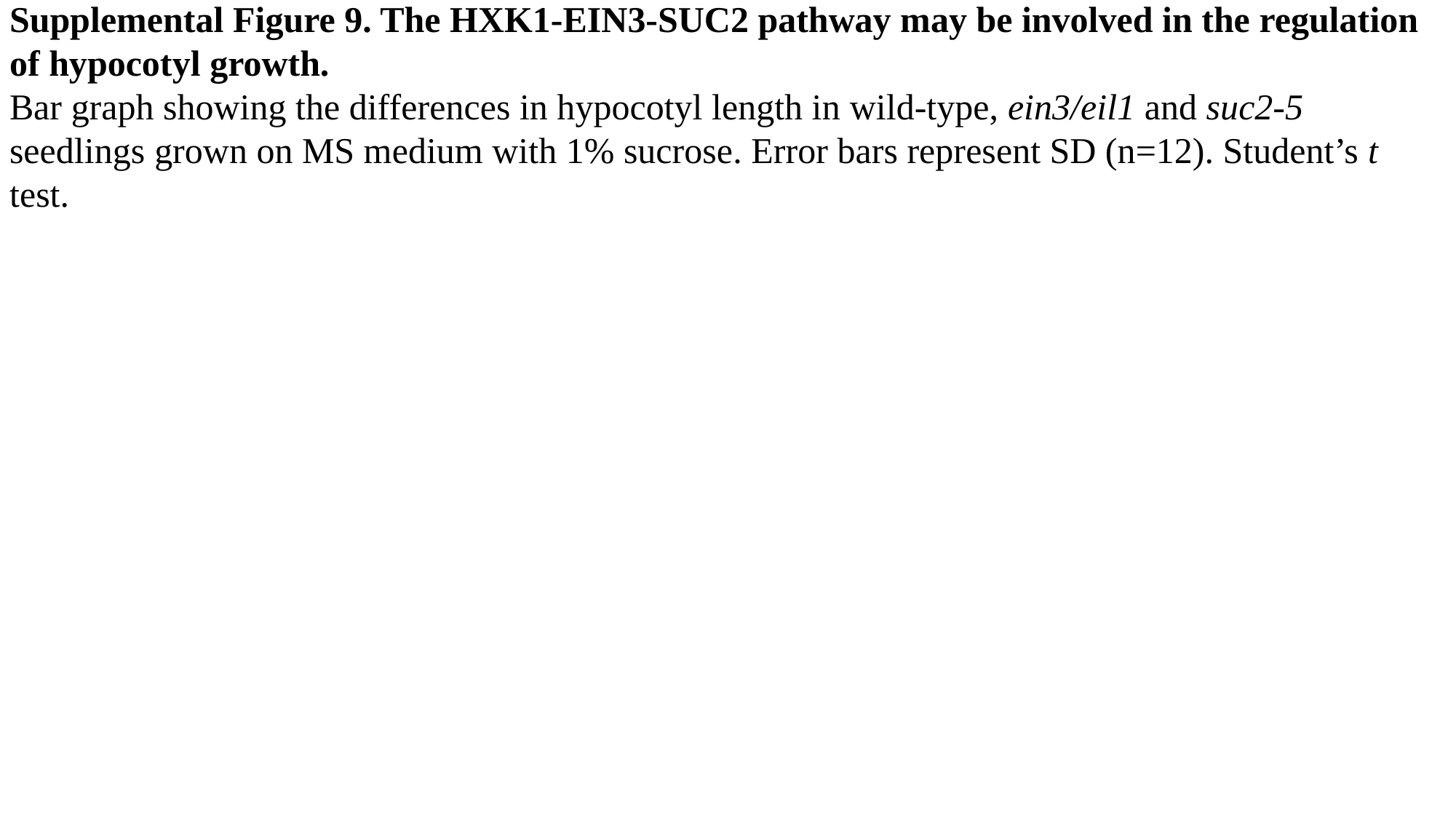

Supplemental Figure 9. The HXK1-EIN3-SUC2 pathway may be involved in the regulation of hypocotyl growth.
Bar graph showing the differences in hypocotyl length in wild-type, ein3/eil1 and suc2-5 seedlings grown on MS medium with 1% sucrose. Error bars represent SD (n=12). Student’s t test.
